# An Excitatory Loop with Astrocytes Contributes to Drive Neurons to Seizure Threshold

**DOI:** 10.1371/journal.pbio.1000352

**Published:** 2010-04-13

**Authors:** Marta Gómez-Gonzalo, Gabriele Losi, Angela Chiavegato, Micaela Zonta, Mario Cammarota, Marco Brondi, Francesco Vetri, Laura Uva, Tullio Pozzan, Marco de Curtis, Gian Michele Ratto, Giorgio Carmignoto

**Affiliations:** 1 Institute of Neuroscience – Consiglio Nazionale delle Ricerche (CNR), University of Padova, Padova, Italy; 2 Department of Experimental Biomedical Sciences, University of Padova, Padova, Italy; 3 National Enterprise for nanoScience and nanoTechnology (NEST), Instituto Nanoscienze CNR, Scuola Normale Superiore, Pisa, Italy; 4 Institute of Neuroscience – CNR, Pisa, Italy; 5 Fondazione Istituto Neurologico Carlo Besta, Milano, Italy; 6 Venetian Institute of Molecular Medicine, Padova, Italy; Stanford University School of Medicine, United States of America

## Abstract

Seizures in focal epilepsies are sustained by a highly synchronous neuronal discharge that arises at restricted brain sites and subsequently spreads to large portions of the brain. Despite intense experimental research in this field, the earlier cellular events that initiate and sustain a focal seizure are still not well defined. Their identification is central to understand the pathophysiology of focal epilepsies and to develop new pharmacological therapies for drug-resistant forms of epilepsy. The prominent involvement of astrocytes in ictogenesis was recently proposed. We test here whether a cooperation between astrocytes and neurons is a prerequisite to support ictal (seizure-like) and interictal epileptiform events. Simultaneous patch-clamp recording and Ca^2+^ imaging techniques were performed in a new in vitro model of focal seizures induced by local applications of N-methyl-D-aspartic acid (NMDA) in rat entorhinal cortex slices. We found that a Ca^2+^ elevation in astrocytes correlates with both the initial development and the maintenance of a focal, seizure-like discharge. A delayed astrocyte activation during ictal discharges was also observed in other models (including the whole in vitro isolated guinea pig brain) in which the site of generation of seizure activity cannot be precisely monitored. In contrast, interictal discharges were not associated with Ca^2+^ changes in astrocytes. Selective inhibition or stimulation of astrocyte Ca^2+^ signalling blocked or enhanced, respectively, ictal discharges, but did not affect interictal discharge generation. Our data reveal that neurons engage astrocytes in a recurrent excitatory loop (possibly involving gliotransmission) that promotes seizure ignition and sustains the ictal discharge. This neuron–astrocyte interaction may represent a novel target to develop effective therapeutic strategies to control seizures.

## Abstract 2

Studies in rodent brain slices suggest that seizures in focal epilepsies are sustained and propagated by the reciprocal interaction between neurons and astroglial cells

## Author Summary

In focal epilepsy, seizures are generated by a localized, synchronous neuronal electrical discharge that may spread to large portions of the brain. Despite intense experimental research in this field, a key question relevant to the human epilepsy condition remains completely unanswered: what are the cellular events that lead to the onset of a seizure in the first place? In various in vitro models of seizures using rodent brain slices, we simultaneously recorded neuronal firing and Ca^2+^ signals both from neurons and from astrocytes, the principal population of glial cells in the brain. We found that activation of astrocytes by neuronal activity and signalling from astrocytes back to neurons contribute to the initiation of a focal seizure. This reciprocal excitatory loop between neurons and astrocytes represents a new mechanism in the pathophysiology of epilepsy that should be considered by those aiming to develop more effective therapies for epilepsies that are not controlled by currently available treatments.

## Introduction

Focal epilepsies are characterized by a condition of neuronal hyperexcitability that is restricted to the epileptogenic region. Focal seizures originate at this region and secondarily spread to distant cortical areas [1]–[5]. Several factors, from ion channel mutations to brain injury, may cause neuronal hyperexcitability changes that sustain an epileptic condition [6]. Yet, the earlier cellular events that initiate a seizure in the first place are still unclear. The understanding of ictogenesis is thus central to the pathophysiology of focal epilepsies and is a requirement to develop new pharmacological therapies for drug-resistant focal epilepsies [7].

In the present study, we specifically address the hypothesis that the activation of a loop between neurons and astrocytes is an early event that contributes to focal seizure initiation. This hypothesis stems from a series of recent studies that reappraised the role of neurons in epileptogenesis and hinted at a possible, direct contribution of astrocytes to the generation of an epileptic discharge. The first clue was the observation that the release of glutamate from astrocytes, elicited by Ca^2+^ oscillations, promotes local synchronous activities in hippocampal neurons by acting on extrasynaptic N-methyl-D-aspartic acid (NMDA) receptors [8]. Studies performed both on brain slices and in vivo showed that during epileptiform activity, the frequency of Ca^2+^ oscillations in astrocytes is significantly increased [9],[10], and it is reduced by anticonvulsant drugs [9]. Moreover, the expression of metabotropic glutamate receptors (mGluRs, mediators of Ca^2+^ oscillations in these cells) in hippocampal astrocytes from animal models of temporal lobe epilepsy was found to be increased [11],[12]. These observations suggest that the excessive neuronal synchronization that characterizes the epileptic discharge might be sustained, at least in part, by an astrocyte hyperactivity. In support of an astrocyte role in epileptiform activities, it has been proposed that the interictal events recorded between seizures might be in some conditions tetrodotoxin (TTX)-resistant and mediated by glutamate release from astrocytes [9]. These findings fuelled a controversial debate on the role of astrocytes in focal epileptogenesis and in the generation of epileptiform discharges [13]–[15].

In the present study, we used different models of epileptic seizures, including a new model of focal seizures, to define the role of astrocytes in the generation of epileptiform activities. We performed simultaneous Ca^2+^ imaging and electrophysiological recordings of epileptic discharges in brain slices and in isolated intact guinea pig brains, focusing on the entorhinal cortex. This experimental approach allowed us to define the timing of astrocyte Ca^2+^ excitability in relation to interictal and ictal discharges. By using different pharmacological tools to affect selectively the Ca^2+^ signal in astrocytes, we also investigated a possible causative role of astrocyte activation in the generation of these epileptic discharges.

We demonstrate here that a recurrent excitatory loop between neurons and astrocytes involving Ca^2+^ elevations in a large number of glial cells is an early event that contributes to the initiation of a focal seizure-like discharge.

## Results

### A Large Number of Astrocytes Are Activated by Ictal, but Not Interictal, Discharges

#### Picrotoxin/zero-Mg^2+^ entorhinal cortex slice model

**Figure 1:**
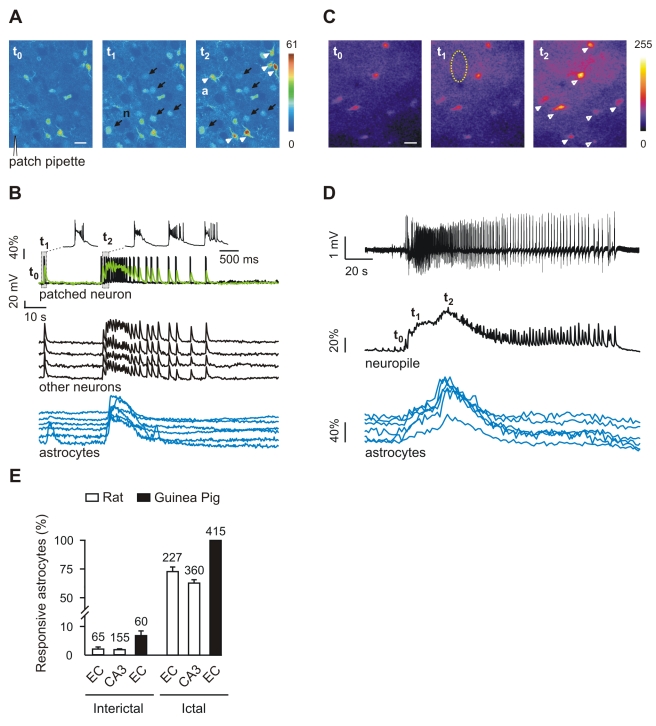
The large majority of astrocytes respond with a Ca^2+^ elevation to ictal, but not interictal, discharges. (A) Ca^2+^ changes, with respect to the basal level (*t*_0_), in neurons (n, black arrows) and astrocytes (a, white arrowheads) during an interictal (*t*_1_) or an ictal (*t*_2_) event after EC slice perfusion with picrotoxin/zero-Mg^2+^. Scale bar represents 20 µm. (B) AP bursts and Ca^2+^ change (green trace) from the patched neuron, and Ca^2+^ changes from other neurons and from astrocytes indicated in (A). Note the large Ca^2+^ rise evoked in astrocytes by ictal (*t*_2_) and not by interictal (*t*_1_) events. (C) 2P-LSM Ca^2+^ imaging in guinea pig EC before (*t*_0_) and during the development (*t*_1_ and *t*_2_) of the ictal discharge induced by arterial perfusion with bicuculline. Scale bar represents 20 µm. (D) Field potential and Ca^2+^ signal changes from neuropile (dashed circle in [C]) and astrocytes (arrowheads in [C]) during the ictal discharge. (E) Mean percentage of interictal- and ictal-activated astrocytes. Numbers over each bar indicate the total number of astrocytes examined. The interictal and ictal events analyzed were, respectively, 130 and 21 in rat CA3, 69 and 15 in rat EC, 347 and 31 in guinea pig EC. Error bars in this and other figures indicate SEM.

In a first series of experiments, we investigated neuron and astrocyte activities in entorhinal cortex (EC) slices during interictal and ictal discharges induced by the gamma-aminobutyric acid (GABA)_A_ receptor inhibitor picrotoxin applied in Mg^2+^-free solution. Slice incubation with the Ca^2+^ dye Oregon Green BAPTA-1 acetoxymethyl ester (OGB1-AM) allowed us to monitor Ca^2+^ signals from both neurons and astrocytes, identified according to morphological and functional criteria [16]–[18] (see also Materials and Methods). Patch-clamp recordings coupled to Ca^2+^ imaging revealed a clear correlation between action potential (AP) bursts and Ca^2+^ changes from the patched neuron during both the brief interictal and the prolonged ictal discharges (Figure 1A and 1B). Ca^2+^ elevations with similar onset and time course were also observed in unpatched neurons simultaneously monitored in the same field (Figure 1A, and other neurons in 1B). These observations demonstrate that the neuronal Ca^2+^ signal reflects faithfully the AP discharge during ictal and interictal discharges and represents a useful tool to i) detect epileptic discharges; ii) mark ictal discharge initiation; and iii) evaluate the extension of underlying neuronal synchronies.

A Ca^2+^ rise was distinctly activated by ictal discharges in most astrocytes, whereas interictal discharges failed to evoke a similar astrocyte activation (Figure 1A and 1B; see also Figure 1E and Video S1), and it increased only the frequency of independent Ca^2+^ oscillations in single astrocytes (Figure S1). In a total of 15 experiments, 73.5±4.0% of the astrocytes present in the recording field (*n* = 227; Figure 1E) were activated by the ictal discharge, and in most of these (57.7%) a Ca^2+^ elevation occurred 1.8±0.2 s after the ictal discharge onset. A similar distinct activation of astrocytes during the ictal event evoked by picrotoxin/zero-Mg^2+^ was observed also in CA3 region from hippocampal slices of both rats (Figure 1E) and pGFAP-EGFP transgenic mice in which astrocytes are labelled by the enhanced green fluorescent protein (EGFP) under the control of the human glial fibrillary acidic protein (GFAP) promoter (unpublished data).

#### Bicuculline-perfused, whole guinea pig brain model

To validate in an intact brain the findings obtained in EC slices, we used the in vitro isolated whole brain from young adult guinea pigs [19] since imaging of the EC is impracticable in vivo. In this preparation, networks responsible for focal ictogenesis in the EC–hippocampus have been analyzed in detail [20],[21]. We simultaneously recorded the extracellular field potential and Ca^2+^ signals by two-photon laser scanning microscopy (2P-LSM) during epileptiform activities induced by arterial application of the GABA_A_ receptor antagonist, bicuculline methiodide. Ca^2+^ signals in neuropile were tightly correlated with the changes in the field potential observed during the seizure discharge and increased in parallel with the appearance of a fast activity at 20–30 Hz that accompanied the onset of the ictal discharge [21],[22] (Figure 1D). As in brain slices, in this close to in vivo condition, seizure-like events regularly evoked Ca^2+^ elevations in astrocytes (Figure 1C and 1D), whereas interictal events failed to activate astrocyte responses (Figure S1). A bar graph summarizes the different response of astrocytes to interictal and ictal discharges in the different models (Figure 1E).

### Astrocyte Activation by the Ictal Discharge Involves Glutamate and ATP

**Figure 2:**
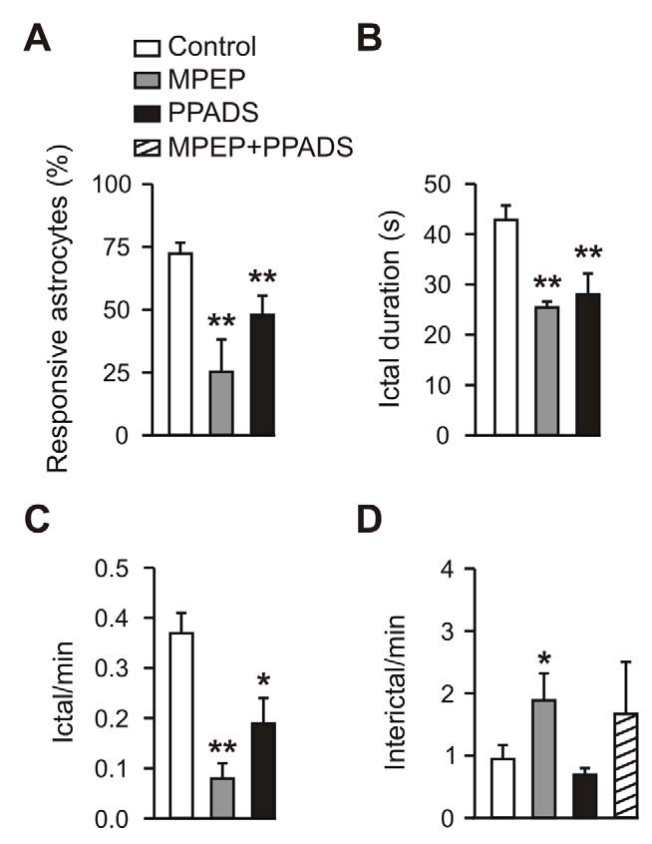
Astrocyte Ca^2+^ signal inhibition does not affect interictal discharges. (A–D) Mean percentage of astrocytes activated by the ictal discharges (A), mean duration (B) and frequency (C) of the ictal discharge, and mean frequency of interictal discharges (D) under different experimental conditions in EC slice preparations. Controls (*n* = 16), MPEP (*n* = 7), PPADS (*n* = 9), and MPEP+PPADS (*n* = 3). A single asterisk (*) indicates *p*<0.05; double asterisks (**), *p*<0.01.

The activation of astrocytes by neuronal activity is mainly mediated by synaptic neurotransmitter release, such as glutamate [16],[23] and ATP [24]. We next asked whether these neuronal signals mediate Ca^2+^ elevations triggered in astrocytes by the ictal discharge. We found that the activation of astrocytes by the ictal discharge was significantly reduced by slice perfusion with either the antagonist of mGlu receptors 2-methyl-6-(phenylethynyl)-pyridine (MPEP), or the antagonist of purinergic (P2) receptors pyridoxal phosphate-6-azophenyl-2′,4′-disulfonic acid (PPADS, Figure 2A). MPEP/PPADS co-perfusion abolished ictal discharges, thus hampering the possibility to clarify whether glutamate and ATP can entirely account for astrocyte activation by the ictal event.

We also found that after slice perfusion with either MPEP or PPADS, the duration and frequency of ictal episodes in neurons were significantly reduced with respect to controls (Figure 2B and 2C), whereas interictal discharges were either unaffected (PPADS and MPEP/PPADS) or increased in frequency (MPEP; Figure 2D). These results clearly show that Ca^2+^ elevations mediated by mGlu and P2 receptors in astrocytes (and neurons) do not have a role in the generation of interictal discharges. Given that MPEP and PPADS block receptors in both neurons and astrocytes, these results also suggest that Ca^2+^ signals activated by these receptors, on one or both cells, may have a role in ictal discharge generation.

### Selective Activation of Astrocytes Favours Ictal Discharge Generation

**Figure 3:**
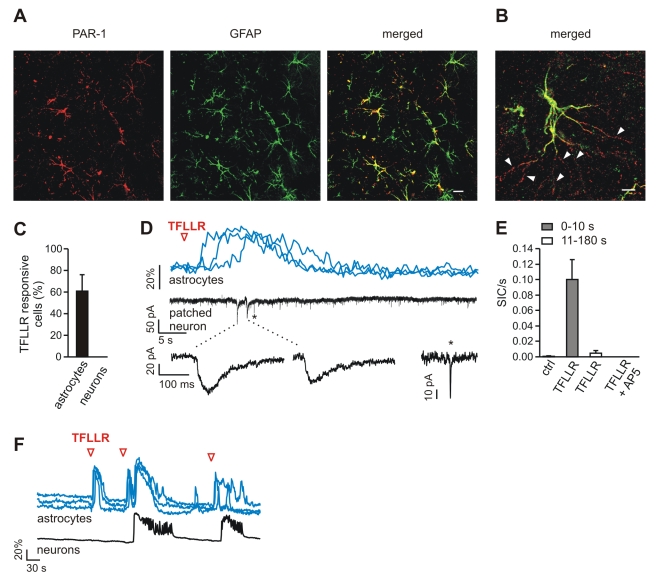
PAR-1 receptor activation in astrocytes favours ictal discharge generation. (A) Images showing PAR-1 (red) and GFAP (green) immunoreactivity and the merged image in EC layers V-VI; 86.7% of GFAP-positive astrocytes were PAR-1 immunopositive. Scale bar represents 20 µm. (B) High magnification of a confocal image showing a merged image of PAR-1 and GFAP immunoreactivity from EC layer V-VI. Note the continuity of PAR-1 punctate signals that are presumably associated with GFAP-negative distal portions of GFAP-positive astrocyte processes (white arrowheads). Scale bar represents 5 µm. (C) Bar graph showing that bath perfusion with TFLLR (10 µM) in the presence of D-AP5 and TTX failed to trigger any Ca^2+^ response in 70 neurons analyzed from three different experiments. (D) Ca^2+^ elevations in astrocytes (top traces) and whole-cell recording of an adjacent neuron (lower trace) after a single TFLLR pulse (indicated by an open red arrowhead) applied in the presence of TTX. The Ca^2+^ elevation in astrocytes is followed by the activation of SICs in the nearby patched neuron. Note the slower kinetics of SICs (insets) with respect to those of a spontaneously occurring fast synaptic miniature event (*). (E) Bar graph reporting the frequency of SICs before and after TFLLR pulses. The large majority of SICs (six of 11) occurred within 10 s of the TFLLR-evoked Ca^2+^ increase in astrocytes. No SICs where observed after TFLLR induced Ca^2+^ elevations in astrocytes in the presence of D-AP5. (F) In the picrotoxin/zero-Mg^2+^ model, astrocyte stimulation by TFLLR (open red arrowhead) was sufficient to evoke an ictal discharge.

We next asked whether astrocyte Ca^2+^ elevations may have a specific role in ictal discharge generation. To investigate this hypothesis, an agonist able to selectively trigger a Ca^2+^ increase in astrocytes should be used. The peptide TFLLR, a PAR-1 thrombin receptor agonist, is preferentially expressed in astrocytes and is known to activate glutamate release in astrocytes [25],[26]. We found that PAR-1 immunoreactivity in the EC was largely associated with the soma and the processes of GFAP-positive astrocytes (Figure 3A). Noteworthy, GFAP-negative PAR-1 puncta appeared in continuity with distal portions of astrocyte processes, where GFAP is barely expressed [27] (Figure 3B). Following TFLLR (10 µM) bath perfusion in the presence of both TTX and D-2-amino-5-phosphonopentanoate (D-AP5), which blocks NMDAR-mediated astrocyte-to-neuron signalling [8],[28], we could not detect any Ca^2+^ change in EC neurons, whereas large Ca^2+^ elevations were observed in astrocytes (Figure 3C).

We next asked whether PAR-1 receptor activation could stimulate the release of glutamate from EC astrocytes, as previously reported for hippocampal astrocytes [25],[26]. We found that Ca^2+^ elevations triggered in EC astrocytes by short pressure pulses applied to a TFLLR-containing pipette (1 mM) were followed by slow inward currents (SICs) in adjacent patched neurons (Figure 3D). Most of the SICs recorded in six of 12 neurons occurred within 10 s (mean delay ± SEM, 1.3±0.3 s) after the TFLLR-induced Ca^2+^ elevations in astrocytes (Figure 3D and 3E). Unlike fast spontaneous synaptic currents (asterisks in Figure 3D), SICs have typical slow kinetics (rise time, 83.0±36 ms, decay time, 451±171 ms; *n* = 13), are insensitive to TTX, and are sensitive to the NMDAR blocker D-AP5 (Figure 3E), as demonstrated in neurons from other brain regions [8],[28]–[30].

In the picrotoxin/zero-Mg^2+^ entorhinal cortex slice model, we then investigated whether selective astrocyte activation enhanced ictal discharge generation. We found that Ca^2+^ elevations triggered in astrocytes by local TFLLR applications were sufficient to shift neurons towards the ictal discharge threshold (Figure 3F; Video S2). To demonstrate the causal link between the ictal discharge and the immediately preceding TFLLR-induced Ca^2+^ increase in astrocytes, we simulated the ictal occurrence by a Monte Carlo procedure. Results from this analysis revealed that in six experiments in which 30 TFLLR applications were performed, 10 of the 15 observed ictal events were correlated at the 0.05 confidence level with a preceding astrocyte Ca^2+^ increase (Figure S2).

These results suggest that when the level of basal excitability and the predisposition of neurons to generate epileptiform discharges is high, as in the picrotoxin/zero-Mg^2+^ model, activation of the NMDAR by astrocytic glutamate could trigger neuronal hyperactivity that is sufficient to generate an ictal discharge. Compelling, although indirect, support for this hypothesis derived from the observation that a short pressure-pulse application of NMDA via an NMDA-containing pipette could also evoke an ictal discharge (Figure S3).

### In the Presence of 4-AP, a Local Application of NMDA Evokes a Focal Ictal Discharge

To further investigate the possible role of astrocytes in seizure initiation, we developed a model of focal seizures alternative to the picrotoxin/zero-Mg^2+^ model. In this latter model, indeed, epileptic activities arise spontaneously and at unpredictable foci [31],[32], and therefore the cellular mechanism of seizure initiation cannot be analyzed accurately.

**Figure 4:**
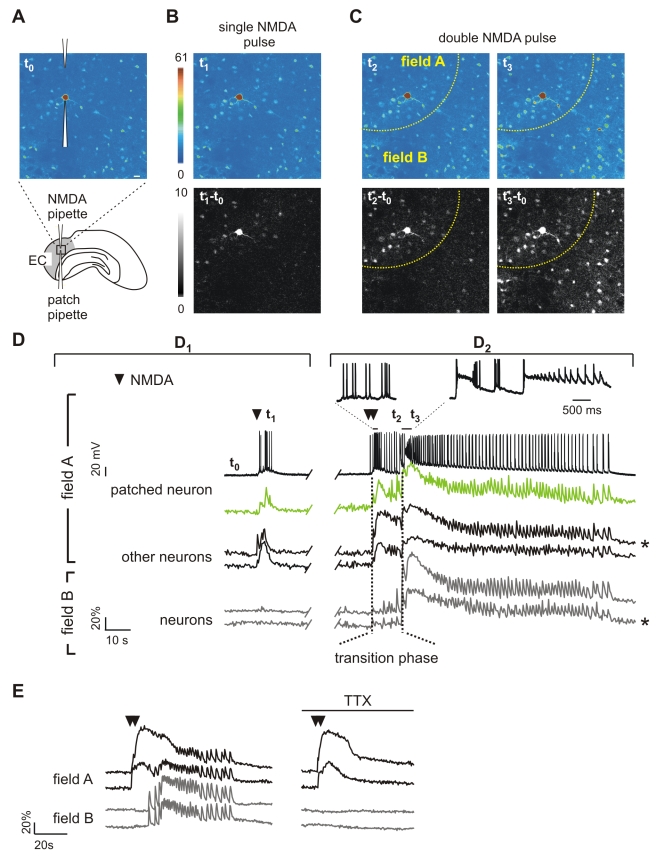
Local neuronal activation evokes ictal discharges. (A) Drawing and pseudocolour image of the Ca^2+^ signal at basal conditions (*t*_0_) of an EC slice showing the NMDA-containing pipette and the patch pipette on a layer V neuron. Scale bar represents 20 µm. (B) Pseudocolour (*t*_1_) and difference image (*t*_1_ − *t*_0_) of local Ca^2+^ changes after a single NMDA pulse. (C) Pseudocolour and difference images of the Ca^2+^ response triggered by a double NMDA pulse. *t*_2_ and *t*_2_ − *t*_0_ illustrate the initial Ca^2+^ elevation in neurons from the region close to the NMDA pipette (field A, dashed yellow line) and in some neurons from the surrounding field B. *t*_3_ and *t*_3_ − *t*_0_ illustrate the Ca^2+^ change in all neurons reflecting the ictal discharge. (D) (D_1_) NMDA-induced AP firing from the patched neuron and Ca^2+^ changes from this (green trace) and other neurons from field A and field B after a single NMDA pulse (indicated by a black arrowhead). (D_2_) AP bursts from the patched neuron (see also insets) and Ca^2+^ changes from this and other neurons from field A and field B during the ictal discharge induced by a double NMDA pulse (black arrowheads). Vertical dashed lines mark the transition phase from the onset of the direct response (left line) to the emergence of the ictal discharge in field B neurons (right line). *t*_0-3_ mark the timing for the images shown in (A–C). *Traces are reported also in Figure 5. (E) TTX blocked the ictal discharge in both field A and B neurons, but not the direct response of field A neurons to a double NMDA pulse (black arrowheads).

In our new model, ictal discharges are reproducibly generated at discrete sites of the EC by focal NMDA applications. Figure 4A reports schematically the positions of an NMDA-containing pipette and an OGB1-containing patch pipette in layer V of the EC. A confocal image of this region under basal conditions (*t*_0_) is also presented. Focal episodes of neuronal hyperactivity are induced in the presence of 100 µM 4-aminopyridine (4-AP) and 0.5 mM Mg^2+^ by short pressure-pulse applications of NMDA via the NMDA-containing pipette. The effect of the NMDA pulse ejection was monitored by simultaneously recording Ca^2+^ signals from neurons and the AP firing from one of the neurons close to the NMDA pipette. Notably, in the submerged chamber experiment used in our study, no spontaneous ictal discharges were observed during 4-AP slice perfusion, whereas under different experimental conditions, such as in interface chamber experiments, epileptiform activities arise spontaneously [2]. As illustrated by the fluorescence change, a single NMDA pulse stimulated a transient Ca^2+^ increase in a limited number of layer V-VI neurons from the region close to the pipette tip, that we termed the *field A* (*t*_1_, Figure 4B; Video S3). This local response is clearly illustrated by the difference image generated by subtracting the fluorescence image captured at basal conditions to that obtained after the NMDA stimulation (*t*_1_ − *t*_0_, Figure 4B). Simultaneous patch-clamp recording and Ca^2+^ imaging revealed that the NMDA stimulus leads to AP burst firing in the patched neuron coupled with a Ca^2+^ elevation in this and the other neurons from field A, but it failed to activate neurons of the surrounding region, which we termed *field B* (Figure 4D_1_).

Two-pulse NMDA stimulation with a 3-s interval evoked a stronger activation of neurons and a transient Ca^2+^ elevation in some of the previously unresponsive neurons from the surrounding field B (*t*_2_ and *t*_2_ − *t*_0_, Figure 4C). The response to the double NMDA pulse evolved into a sustained plateau with superimposed Ca^2+^ spikes correlated with AP bursts typical of an ictal discharge, i.e., the cellular equivalent of a seizure [2] (Figure 4D_2_, see also Figure 4C, *t*_3_). The ictal discharge was characterized by Ca^2+^ spikes from unpatched neurons in both field A and field B, highly synchronized with the AP bursts (Figure 4D_2_; Video S4). The recruitment of neurons in field B that underlines the spreading to this region of the ictal discharge is also clearly illustrated by the difference image *t*_3_ − *t*_0_ (Figure 4C). The time window between the double NMDA pulse and the Ca^2+^ elevation that occurs synchronously in both field A and B neurons represents a transition phase during which the ictal discharge develops in field A. In the presence of TTX, the ictal discharge in both field A and B neurons was abolished, whereas the initial response of field A neurons was unaffected (Figure 4E). The size of the cortical region occupied by neurons that respond directly with a transient Ca^2+^ rise to a double NMDA pulse applied in the presence of TTX was 369±17 µm. Notably, the number of neurons in this response (56.5±7.2) is underestimated since it comprises only neurons activated by NMDA in a single focal plane. These results demonstrated that i) AP-mediated events secondary to the initial activation of field A neurons are crucial for ictal discharge maturation; and ii) the activation of neurons from field B and the generation of the ictal discharge was not due to a delayed diffusion of NMDA. Paired recordings from two pyramidal neurons (one in field A and the other in field B) confirmed that similar ictal discharges were regularly evoked in field A and B by successive double NMDA pulses (Figure S4).

According to results obtained from 14 experiments, no failures were observed in a total of 101 double NMDA pulse stimulations, and the mean duration of the ictal discharge repetitively evoked by these stimulations was reproducible over long time periods (up to 60 min, Figure S4). By applying successive double NMDA pulses in the presence of TTX, no NMDA-mediated Ca^2+^ elevations were detected in field B neurons, whereas the number of field A neurons activated directly by NMDA and the amplitude of their Ca^2+^ response were found to be unchanged over the same time period (Figure S4).

Ictal discharges could be evoked also by two single NMDA pulses applied at two different sites, either simultaneously or in succession. Intervals of 3 or 5 s were successful, but not an interval of 10 s. To be effective, the two pipette tips should be positioned close enough to allow a large spatial overlapping of the two pulses. Only in this overlapping region were neurons strongly activated by the two NMDA pulses. Notably, if the distance between the two pipette tips was 172±30.2 µm (*n* = 5) (a value similar to the mean radius of the field A directly activated by double NMDA pulses), the two single NMDA pulses regularly evoked an ictal discharge. If the distance of the two pipette tips was 220±38.5 µm, no ictal discharges could be evoked.

Altogether, these data show that an episode of activity evoked in a group of neurons by local NMDA applications creates an initiation site for a seizure-like discharge that secondarily involves adjacent neuronal populations. They also demonstrate that our model is highly reliable since comparable ictal discharges can be evoked by repetitive stimulations applied to the same restricted site. Notably, in contrast to the picrotoxin/zero-Mg^2+^ model, in the 4-AP model, single NMDA pulses failed to trigger focal ictal discharges, suggesting different thresholds for seizure generation in these two models (see Discussion).

### The Development of the Ictal Discharge Is Accompanied by Astrocyte Activation

**Figure 5:**
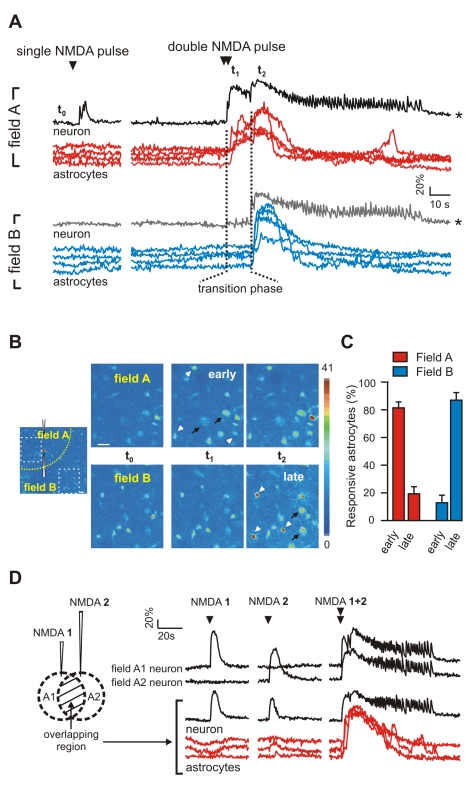
The ictal discharge generation is accompanied by Ca^2+^ elevations in astrocytes. (A) Ca^2+^ changes of a field A neuron, a field B neuron, and astrocytes in field A and field B from the same experiment illustrated in Figure 4A–4D. The single NMDA pulse fails to activate astrocytes (red and blue traces, left), whereas the double NMDA pulse evokes a large astrocyte Ca^2+^ rise that in field A (red traces, right) is associated with the initial development of the ictal discharge, whereas in field B (blue traces, right), the rise follows it. Vertical dashed lines mark the transition phase. *Traces from Figure 4 are shown for comparison. (B) Image at high magnifications (dashed boxes in left panel) illustrating the early Ca^2+^ increase that in field A astrocytes occurs during the transition phase (*t*_1_) and the late Ca^2+^ increase that in field B astrocytes occurs after the ictal discharge (*t*_2_). Arrows and arrowheads mark neurons and astrocytes, respectively. Scale bars represent 20 µm. (C) Percentage of field A astrocytes (13 experiments, 262 responsive astrocytes) and field B astrocytes (12 experiments, 187 responsive astrocytes) that displayed an early or a late Ca^2+^ increase. (D) Left, drawing showing the position of the two NMDA-containing pipettes (NMDA 1 and NMDA 2) and the overlapping region of activation by both NMDA pulses. Right, Ca^2+^ changes from a field A1 neuron, a field A2 neuron, and from a neuron and the astrocytes in the overlapping region in response to single NMDA pulses applied to either one or both pipettes. Single NMDA pulses applied simultaneously to both pipettes evoke a large astrocyte Ca^2+^ rise in the overlapping region and the ictal discharge.

We next investigated astrocyte activities during the development of focal ictal discharges. We observed that shortly after the initial neuronal response to a double NMDA pulse, a large Ca^2+^ elevation occurred almost simultaneously in the large majority of field A astrocytes (Figure 5A, red traces; Video S4). Similar Ca^2+^ elevations in these astrocytes were never observed during the neuronal response to a single NMDA pulse. In 13 experiments, a mean of 17.4±3.5 out of 20±3.1 responsive astrocytes in field A displayed an early Ca^2+^ elevation during the transition phase. As a mean, astrocyte activation in field A occurred 4.8±1.1 s before field B neurons were recruited into the ictal discharge. Most of the astrocytes in field B were activated later, i.e., after the invasion of the ictal discharge into this region (Figure 5A, blue traces; Video S4). High-magnification images in Figure 5B illustrate “early” and “late” Ca^2+^ changes of astrocytes from field A and B, respectively. The mean percentage of astrocytes from field A and B displaying “early” and “late” responses is reported in Figure 5C. Notably, when the ictal discharge was evoked by two single NMDA pulses applied at two distinct sites (Figure 5D), most astrocytes from both the field of spatial overlapping of the two pulses and the immediately surrounding regions (fields A1 and A2) displayed a similar early Ca^2+^ elevation (85.6±5.4%), whereas most astrocytes from the surrounding regions (the fields B) showed a late activation (71.6±5.4%). Noteworthy is that astrocytes failed to be similarly activated by each single NMDA pulse alone (Figure 5D).

We next asked whether the initial Ca^2+^ elevation in astrocytes (and neurons) from field A spread to other astrocytes (and neurons) in the surrounding regions through a concentric wave of activation centred on the NMDA pipette. We found that the Ca^2+^ response of astrocytes as well as the recruitment of neurons into the ictal discharge is more consistent with a process of modular recruitments rather than with a propagation of a concentric wave of activity (Figure S5).

Astrocyte activation was largely due to AP-mediated neurotransmitter release since 70.4±8.3% (*n* = 143, 5 experiments) of the field A astrocytes, activated by a first double NMDA pulse, failed to respond to a second double NMDA pulse applied in the presence of TTX. The Ca^2+^ rise in still-responsive astrocytes displayed slow kinetics and were of small amplitude (Δ*F*/*F*_0_, 64.1±3.6 before and 29.0±2.2 after TTX; *n* = 41; *p*<0.001). This residual astrocyte response in TTX could be due either to neurotransmitter release mediated by activation of presynaptic NMDA receptors [33] or to the direct activation by NMDA of NMDA receptors that may be present on astrocytes [34],[35].

The results from these experiments indicate that the development of a focal ictal discharge is accompanied by Ca^2+^ elevations in astrocytes.

### Selective Inhibition of Astrocytes Impairs Ictal Discharge Generation

**Figure 6:**
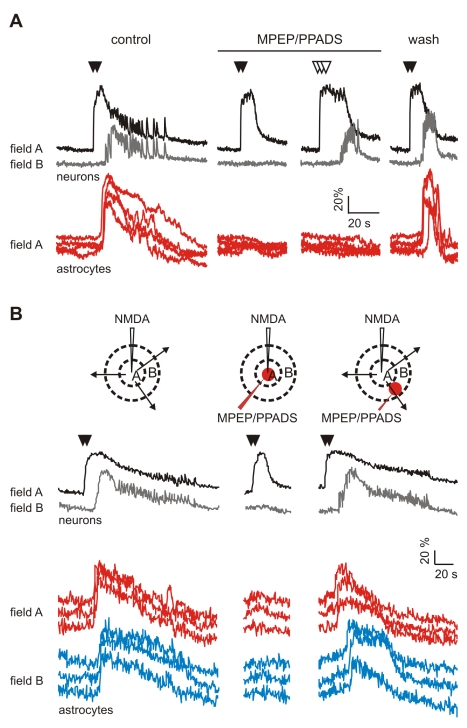
Inhibition of field A astrocytes by MPEP/PPADS impairs ictal discharge generation. (A) Ca^2+^ signal from a field A neuron, a field B neuron, and field A astrocytes in response to repetitive episodes of NMDA stimulation (black arrowheads). The NMDA stimulation that evoked an ictal discharge became ineffective after blocking the astrocyte response by bath perfusion with MPEP and PPADS. An ictal discharge could be recovered by increasing the number of NMDA puffs (white arrowheads). A double NMDA pulse evoked both astrocyte activation and the ictal discharge after inhibitor washout. (B) Top, drawings of field A and B illustrating different experimental conditions. The three black arrows symbolize the spreading of a focal ictal discharge. The red spots indicate the region of local MPEP/PPADS applications. Bottom, Ca^2+^ signal from a field A neuron, a field B neuron, field A astrocytes, and field B astrocytes in response to repetitive episodes of NMDA stimulation (black arrowheads). The double NMDA pulse that evoked an ictal discharge became ineffective after local MPEP/PPADS application to field A, but not to field B. Note also the absence of “early” responsive astrocytes after MPEP/PPADS applications to field A, whereas “late” responsive astrocytes are only slight changed after MPEP/PPADS applications to field B.

If this early Ca^2+^ elevation in astrocytes is not a mere consequence of neuronal activity and has, instead, a causative role in ictal discharge generation, its inhibition should reduce the ability of NMDA to trigger an ictal discharge. To address this hypothesis, we first bath applied MPEP and PPADS (*n* = 4) and found that the direct activation of neurons by a double NMDA pulse was unchanged, but early activated astrocytes were reduced to 4.6±2.6% of controls. Under these conditions, the generation of the ictal discharge in field A and the subsequent recruitment of neurons into the epileptic discharge in field B were inhibited (Figure 6A). The ictal discharge recovered after washout of the antagonists and the reappearance of the associated Ca^2+^ elevation in astrocytes. Interestingly, a stronger neuronal stimulation obtained by increasing the number of successive NMDA puffs evoked an ictal discharge, although of short duration, even in the presence of MPEP/PPADS and without a recovery of astrocyte Ca^2+^ signals (Figure 6A).

We also found that the NMDA-induced ictal discharge was blocked after inhibition of the early responsive astrocytes in field A by MPEP/PPADS applied locally to this region (Figure 6B; *n* = 4). Ictal discharge recovery was regularly observed 5–10 min after cessation of the MPEP/PPADS pulses. In contrast, applications of MPEP/PPADS to a limited sector of field B failed to affect the spread to field B of the ictal discharge generated in field A (*n* = 4). However, it is noteworthy that the Ca^2+^ elevations in astrocytes from this sector were poorly affected (Figure 6B).

Given that MPEP and PPADS are not selective antagonists of Ca^2+^ signals in astrocytes, to provide a direct evidence for a causal link between Ca^2+^ elevations in field A astrocytes and ictal discharge generation, we inhibited Ca^2+^ signals in these astrocytes selectively by introducing the Ca^2+^ chelator 1,2-bis(o-aminophenoxy)ethane-N,N,N',N'-tetraacetic acid (BAPTA; 50 mM) into individual astrocytes through a patch pipette [36].

**Figure 7:**
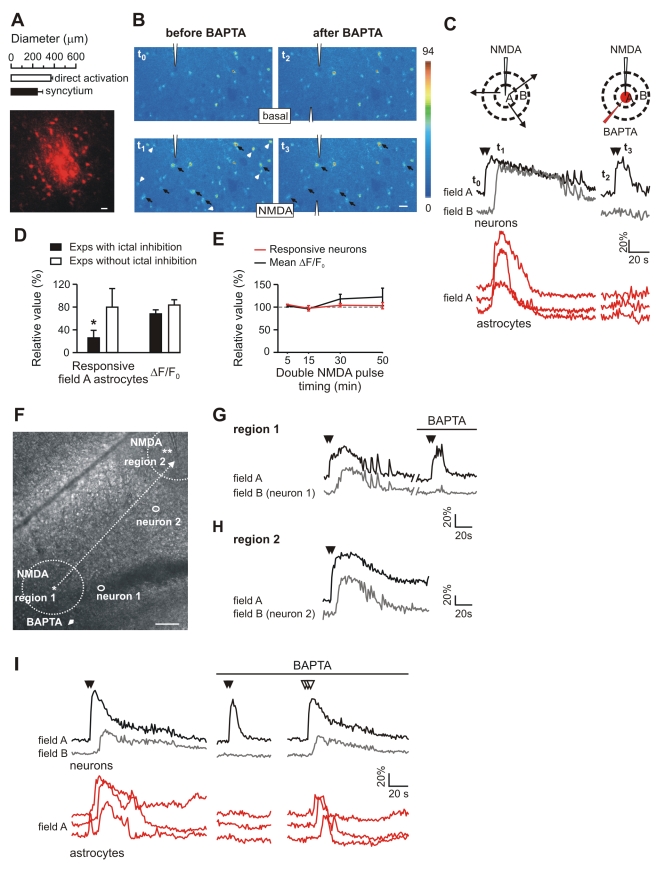
Selective inhibition of astrocytes impairs ictal discharge generation. (A) Maximal projection of a Texas Red–labelled astrocyte syncytium after patching a single astrocyte with a Texas Red–containing pipette in an EC slice. Top, bar graph reporting the mean size of the labelled astrocyte syncytium (*n* = 5) and of the region of neurons directly activated by NMDA (*n* = 4). Scale bar represents 20 µm. (B) Images from an EC slice illustrating the Ca^2+^ signal in neurons and astrocytes from field A before and after BAPTA spreading in the astrocyte syncytium, at rest (*t*_0_ and *t*_2_, respectively) and after the NMDA stimulation (*t*_1_ and *t*_3_, respectively). Responsive neurons (black arrows) and astrocytes (white arrowheads) are indicated. Note the absence of astrocyte responses after BAPTA (*t*_3_). Scale bar represents 20 µm. (C) Ca^2+^ changes from some of the neurons and astrocytes indicated in (B). Also reported is the Ca^2+^ change of the ictal discharge from a field B neuron not present in the image (gray trace). The NMDA stimulation that can evoke an ictal discharge becomes ineffective after inhibition of the astrocyte Ca^2+^ signal in field A by BAPTA (red traces). (D) Change in the number of early responsive field A astrocytes and in the amplitude of the Ca^2+^ increase activated by a double NMDA pulse applied after BAPTA spreading in the astrocyte syncytium, expressed as percentage of control values, i.e., before BAPTA dialysis, in the experiments in which BAPTA inhibited, or failed to inhibit, the ictal discharge. One-sample Student *t*-test, an asterisk (*) indicates *p*<0.05. (E) Number of responsive neurons and amplitude of the Ca^2+^ signal after repetitive double NMDA pulses applied in the presence of TTX during BAPTA spreading in the astrocyte syncytium (*n* = 3), expressed as relative values with respect to measurements performed before BAPTA spreading. (F) Differential interference contrast image of an EC slice showing the BAPTA-containing pipette (arrowhead) and the first location of the NMDA pipette (asterisk in region 1) that was used to trigger the first ictal discharge. The NMDA pipette was then moved to region 2 and its tip is indicated by the two asterisks. Dashed circle indicates the field A in each region. The small circles mark the position of a field B neuron in region 1 (neuron 1) and in region 2 (neuron 2). Scale bar represents 100 µm. (G) Double NMDA pulse–induced ictal discharge in field A and B neurons in region 1 and its failure 50 min after BAPTA spreading in the astrocyte syncytium. (H) Recovery of the ictal discharge evoked by a double NMDA pulse after moving the NMDA pipette to region 2. (I) Ca^2+^ changes in field A and B neurons and field A astrocytes illustrating the inhibition of the ictal discharge by BAPTA in a different EC slice and its recovery after increasing the NMDA stimulation to three pulses (white arrowheads). Note that after increasing the NMDA stimulation, astrocyte activation partially recovers, but the response is delayed and develops after the emergence of the ictal discharge in field B neurons.

First, we indirectly evaluated BAPTA spreading in the astrocyte syncytium by patching single EC astrocytes with a Texas Red-containing pipette. We counted 31±7 red-labelled astrocytes in an area of 242±50 µm in diameter (Figure 7A). This value is close to the size of the cortical region occupied by neurons that respond directly with a transient Ca^2+^ rise to a double NMDA pulse applied in the presence of TTX (Figure 7A). In subsequent experiments, before patching a field A astrocyte with a BAPTA-containing pipette, a double NMDA pulse was applied to trigger an ictal discharge (Figure 7B and 7C). In five out of nine BAPTA experiments, a double NMDA pulse applied 50 min after BAPTA diffusion in the astrocyte syncytium failed to activate both the Ca^2+^ elevations in astrocytes and the ictal discharge (Figure 7B and 7C). Notably, in these five experiments, the response of early activated field A astrocyte was strongly reduced with respect to that observed before BAPTA (Figure 7D). In these experiments, we addressed the contribution of astrocytes in the activation of neurons during the transition phase. In the presence of BAPTA, which specifically inhibited Ca^2+^ signals in field A astrocytes, the number of recruited neurons upon the double NMDA pulse was 33.1±3.2% lower than in controls (*p*<0.05). Such a reduction is unlikely due to experimental variability in the intensity of the NMDA stimulation since the number of neurons activated and the amplitude of their Ca^2+^ responses to successive double NMDA pulse stimulations (as measured in the presence of TTX) were unchanged over a 50-min period (Figure S4). These observations indicate that the recruitment of neurons into the ictal discharge is also mediated by the early activated astrocytes that signal back to neurons.

In the four experiments with BAPTA in which the ictal discharge was preserved, most of the astrocytes in field A still displayed an early Ca^2+^ response, suggesting a defective diffusion of BAPTA in the astrocyte syncytium in these experiments (Figure 7D). These data provide a plausible explanation for the lack of inhibition of the ictal discharge in these BAPTA experiments.

In a number of different control experiments, we found that i) two subsequent double NMDA pulses applied before and 50 min after patching either a neuron (*n* = 8) or an astrocyte (*n* = 4) with a pipette not containing BAPTA always evoked comparable ictal discharges, indicating that such a long time interval does not affect the ability of a double NMDA pulse to trigger an ictal discharge; ii) double NMDA pulses regularly evoked an ictal discharge even after 50 mM BAPTA was puffed directly over the neurons for 2 min via a pipette (*n* = 4), indicating that a leakage of BAPTA, putatively occurring during astrocyte seal formation, cannot account for the ictal discharge inhibition observed in the BAPTA experiments; iii) successive double NMDA pulses applied in the presence of TTX over a period of 50 min, while patching single astrocytes with a BAPTA-containing pipette, evoked an unchanged response in neurons (Figure 7E), demonstrating that the direct response of neurons to NMDA is not affected after BAPTA-mediated inhibition of astrocyte Ca^2+^ signals.

We next asked whether the late activation of astrocytes in field B contributes to the spreading of the ictal discharge. After patching individual field B astrocytes with a BAPTA-containing pipette, we observed that the ictal discharge evoked in field A by a double NMDA pulse still invaded field B and further propagated to the adjacent region, whereas the activation of field B astrocytes was drastically affected both in terms of Ca^2+^ signal amplitude (−56.6±2.4%, *p*<0.001) and kinetics (time to peak, 2.6±0.4 s and 15.2±3.3 s, before and after BAPTA, respectively; *p*<0.001; Figure S6).

As a further control for the specificity of the BAPTA effect, we demonstrated that the ictal discharge inhibition by BAPTA was spatially restricted. After the astrocyte syncytium in region 1 was loaded with BAPTA, a double NMDA pulse stimulation close to the BAPTA-loaded region failed to trigger an ictal discharge, whereas the same NMDA stimulation applied ∼500 µm away from region 1 readily evoked an ictal discharge (region 2, Figure 7F–7H).

The ictal discharge blocked after the BAPTA-mediated inhibition of field A astrocytes was recovered in two of three experiments by applying a stronger stimulation of neurons, such as a triple NMDA pulse (Figure 7I; white arrowheads). Notably is that under these conditions, astrocytes recovered a Ca^2+^ response that was, however, delayed and of reduced amplitude with respect to that without BAPTA. These results are consistent with the hypothesis that the astrocyte contribution to ictal discharge generation is not an absolute requirement and can be bypassed by a stronger stimulation of neurons, as already suggested by the results obtained in MPEP/PPADS experiments.

Taken together, the results of these series of experiments confirm the reliability of the double NMDA pulse paradigm in evoking an ictal discharge over long time periods and, on the other hand, validate the selective inhibition of astrocyte Ca^2+^ signals by intracellular BAPTA application.

### Selective Activation of Astrocytes Favours the Generation of Focal Ictal Discharges

**Figure 8:**
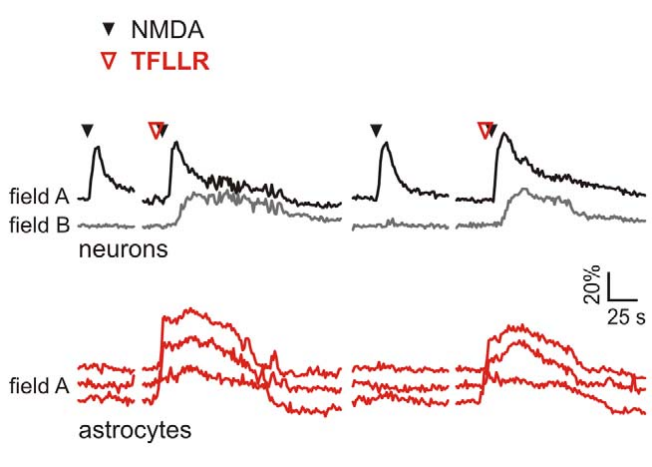
Selective astrocyte activation favours focal ictal discharges. In the 4-AP model, single NMDA pulses evoke the ictal discharge only when an astrocyte Ca^2+^ increase is induced by TFLLR.

If inhibition of Ca^2+^ signals in astrocytes can block the generation of a focal ictal discharge, it would be expected that direct astrocyte stimulation promotes ictal discharges. In the experiments that addressed this hypothesis, we took advantage of the finding that none of the 48 single NMDA pulses performed in the 4-AP ictogenic model could generate an ictal event. Single NMDA pulses that repetitively failed to trigger an ictal discharge became effective when they were coapplied with TFLLR (Figure 8). We found that a single NMDA pulse coupled with TFFLR, evoked an ictal event in six of nine trials from a total of three experiments. In these experiments, we also found that the number of neurons activated by the NMDA/TFLLR coapplication during the transition phase was higher than that activated by NMDA alone (mean increase, +119.3±16.3%; *n* = 6; *p*<0.001). These data confirm that the contribution of astrocytes in the recruitment of neurons can be critical for the generation of the ictal discharge.

## Discussion

In brain slice models of seizures, the ictal discharge is proposed to initiate at focal brain sites by asynchronous neuronal hyperactivities that progressively recruit adjacent neurons into a synchronous discharge [1],[3]–[4]. In our study, we found that neuronal hyperactivities at these restricted brain sites are accompanied by Ca^2+^ elevations in a large number of astrocytes that contribute to drive neurons towards seizure threshold.

**Figure 9:**
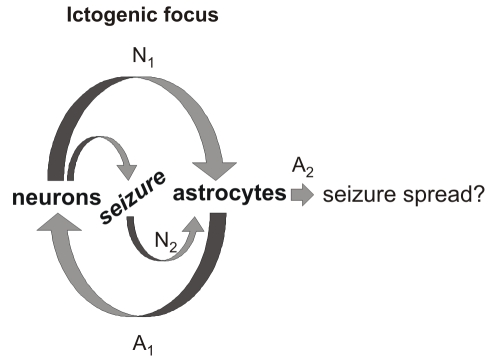
Neuron–astrocyte loop in ictal discharge generation. Schematic of the sequence of events in the recurrent neuron–astrocyte excitatory loop that develops at a restricted brain site to generate seizures. N_1_ and N_2_, neuron-to-astrocyte signalling; A_1_ and A_2_, astrocyte-to-neuron signalling.

The focal ictogenesis in our model is schematically illustrated in Figure 9. This process starts with an isolated episode of local neuronal hyperactivity that triggers a large and synchronous Ca^2+^ elevation in closely associated astrocytes (N_1_). These activated astrocytes signal back to neurons (A_1_) favouring the recruitment of neurons into a coherent activity that underlines the hypersynchronous ictal discharge. This event, in turn, triggers a second activation of astrocytes (N_2_). The secondary astrocyte activation may then contribute to sustain the ictal discharge (A_2_). This sequence of events represents a recurrent neuron–astrocyte excitatory loop that drives neurons towards the ictal discharge threshold.

Since our slice experiments were performed mainly in young animals, the role of astrocytes to seizure generation may be restricted to the immature brain. Although additional experiments are necessary to clarify this important issue, the ability of astrocytes to release glutamate and activate neuronal SICs in slices from young adult rats [8],[28],[37],[38] suggests that astrocyte-to-neuron signalling may contribute to seizure initiation also in the adult brain.

### The Early Ca^2+^ Elevation in Astrocytes Has a Causative Role in Ictal Discharge Initiation

In EC slices perfused with the proconvulsant agent 4-AP in low Mg^2+^ conditions, we found that a synchronous Ca^2+^ elevation in a high number of astrocytes occurred along with the development of the ictal discharge evoked by a local NMDA application. This response was largely TTX sensitive, indicating that astrocytes were activated by AP-mediated neurotransmitter release. Most importantly, the early astrocyte activation was a crucial step in the generation of ictal discharges. Indeed, when Ca^2+^ elevations in field A astrocytes were inhibited by BAPTA, the episode of neuronal hyperactivity induced by NMDA failed to generate an ictal discharge. According to results obtained from different control experiments, the effect of BAPTA on ictal discharge generation was specifically linked to the inhibition of astrocyte Ca^2+^ signals.

The Ca^2+^ elevations in astrocytes are associated with the release of gliotransmitters, such as glutamate [39]–[41] and D-serine [42], that modulates neurotransmitter release [24],[43],[44], triggers AP firing in neurons [10], and promotes local neuronal synchrony [8],[28]. Ca^2+^-dependent release of D-serine from astrocyte activated by Schaffer collateral stimulation has been also recently shown to be crucial for the potentiation of synaptic transmission in the CA1 hippocampal region [45]. As previously reported in the hippocampus [25],[26], we show here that Ca^2+^ elevations stimulated in EC astrocytes by the PAR-1 receptor agonist, TFLLR, triggers glutamate release in these cells and, in turn, NMDA receptor–mediated SICs in neurons. The activation of neurons by gliotransmission can thus account for the finding that a single NMDA pulse, ineffective per se, was able to trigger the ictal discharge if coupled with the direct stimulation of a Ca^2+^ rise in astrocytes by TFLLR. Data analysis of these experiments revealed that the number of neurons activated after NMDA/TFLLR coapplication was higher than that activated after NMDA alone.

These results suggest that when an episode of hyperactivity in a group of neurons consistently engages nearby astrocytes, a larger population of neurons is recruited into a coherent activity. If this feedback signal operates on a brain network prone to seizures, it contributes to drive neurons towards the ictal discharge threshold. The initiation site is thus represented, not only by the neurons activated by NMDA, but also by those that are secondarily activated in a recruitment process that involves astrocytes. Consistent with this view is our finding that when a double NMDA pulse (that successfully evoked an ictal discharge) was applied either after BAPTA was introduced in the astrocyte syncytium or after local applications of MPEP/PPADS to the site of activation, astrocytes were poorly activated, fewer neurons were recruited, and no ictal discharge was evoked. Further support for this conclusion derives from the experiments with a single NMDA pulse delivered from two pipettes positioned at different distances. These experiments revealed that an ictal discharge could be evoked when astrocytes from the region of overlapping neuronal activation were activated. When the pipette tips were more distant, the overlapping region was reduced, astrocytes were poorly activated, and no ictal discharge was evoked.

Distinct subpopulations of astrocytes might differently contribute to modulate neuronal hyperactivity in the epileptogenic region, possibly by releasing in addition to glutamate, ATP, and other neuroactive signals, e.g., GABA, through a Ca^2+^-dependent or -independent mechanism [46],[47]. Given that inhibitory interneurons have been reported to restrain the recruitment of neurons during the development of the ictal discharge [4],[48], an opposite action of astrocytes in this process might involve a distinct inhibition of interneurons by GABA released from astrocytes. Indirect support for this possible astrocyte action derives from the observation that GABA released from astrocytes can, indeed, result in a long-lasting inhibition of inhibitory granule cell activity in the olfactory bulb [37]. Whether a similar signalling between a subpopulation of GABA-releasing astrocytes and interneurons may be involved in ictal discharge initiation in the EC represents an interesting question to be addressed in future studies.

Episodes of focal seizures can arise in a nonepileptic tissue due to genetic causes or as a consequence of various brain damage. These may lead to status epilepticus (SE), a condition of persistent seizures, and evolve into chronic epilepsy after a latent period of epileptogenesis. Our results were obtained in nonepileptic brain tissue and provide evidence for the contribution of astrocytes in the initiation of seizure during SE. Therefore, whether astrocytes contribute also to seizure initiation in chronic epilepsy is, at present, unknown and should be appropriately investigated in chronic epilepsy models. However, results from a recent in vivo study showed that astrocytes, which exhibited long-lasting Ca^2+^ oscillations during SE, contributed to the neuronal death that characterizes chronic epilepsy [38]. This effect was due to astrocytic glutamate that activated neuronal NMDARs, possibly favouring seizure generation. It is also worth underlining that in the epileptic brain tissue, astrocytes undergo significant changes in their physiological properties that may result in decreased glutamate uptake, altered extracellular K^+^ buffering capacities, and activation of inflammatory pathways [49],[50]. All these changes may contribute to the increased neuronal network excitability that characterizes the epileptic brain.

### The Astrocyte Contribution to Ictal Discharge Generation Depends on Neuronal Excitability Levels

The efficacy of astrocyte stimulation in evoking an epileptic discharge was different in the two models used in the present study, probably because of differences in their intrinsic neuronal predisposition to ictal discharge generation. As suggested by the presence of recurrent spontaneous epileptic discharges, the picrotoxin/zero-Mg^2+^ model can be considered, indeed, a model with a low-threshold for epileptic discharges. In this model, a single NMDA pulse triggered synchronous activity in a number of neurons sufficient to reach the ictal discharge threshold, and a single stimulation of astrocytes was also sufficient to trigger an ictal discharge. As suggested by the absence of spontaneous epileptic events, the 4-AP model has a higher threshold for epileptic phenomena. In this model, seizure discharges could be triggered by a more prolonged and intense episode of neuronal activity induced by a double NMDA pulse, and not by single NMDA or TFLLR pulses. An ictal discharge could be also evoked when a single NMDA application (ineffective per se) was coupled with TFLLR-mediated astrocyte activation. Furthermore, the reduction in astrocyte Ca^2+^ signals blocked the ictal discharge in the 4-AP model, but not in the picrotoxin/zero-Mg^2+^ model. These data demonstrate that experimental manipulations of the astrocyte Ca^2+^ signals can influence neuronal recruitment and thus affect, in concert with the level of neuronal activity, the likelihood of ictal events.

As revealed by results from both BAPTA and MPEP/PPADS experiments, when the astrocyte contribution was reduced by inhibiting Ca^2+^ signals in these cells and the double NMDA pulse consequently failed to evoke an ictal discharge, we could recover an ictal discharge by applying a more intense NMDA stimulation. By activating directly a larger number of neurons, this higher stimulus evokes a level of correlated activity that is sufficient for seizure-like discharge generation, bypassing the astrocyte contribution in the recruitment process. Thus, astrocyte activation is not an absolute requirement for ictal discharge generation.

However, astrocytes respond readily to synaptic activity with Ca^2+^ oscillations [16],[23],[51], and the frequency of these oscillations increases in parallel with an increased neuronal activity [16]. In vivo studies also revealed that sensory stimuli can evoke distinct Ca^2+^ elevations in astrocytes confirming the strict association between neuron and astrocyte activities [52]–[55]. Thus, pathological hyperactivities in neurons [6] should be regularly accompanied by an increased astrocyte activity. In support of this view, studies in brain slices showed that chemically induced epileptiform activity causes a sustained increase in astrocyte Ca^2+^ signalling [9],[10], and in vivo studies reported a long-lasting hyperactivity of astrocytes after pilocarpine-induced SE [38]. It is conceivable that a pathological hyperexcitability that predisposes neurons to seizure discharges may originate from abnormalities in the neuron–astrocyte network activity, whatever the origin of the initial dysfunction might be. As we showed here, depending on the different level of excitability in neurons, the astrocyte contribution varies, but it can even be crucial for ictal discharge generation.

### Ictal, but Not Interictal, Discharges Activate a Secondary Astrocyte Ca^2+^ Elevation

In our 4-AP slice model, a second Ca^2+^ elevation even of larger amplitude than that early evoked by the double NMDA pulse, occurred in astrocytes in both field A and field B. This delayed activation of astrocytes was observed also after the spontaneously occurring ictal discharges in the picrotoxin/zero-Mg^2+^ model in both rats and pGFAP-EGFP transgenic mice, as well as in other models such as the 4-aminopyridine/picrotoxin and high-potassium models (unpublished data). Most importantly, this observation was validated in the intact guinea pig brain preparation, a well-characterized model of EC–hippocampus focal ictogenesis [20],[21]. In this close to in vivo preparation, the development of the ictal discharge was regularly accompanied by a Ca^2+^ elevation in virtually all astrocytes present in the recording field, whereas large-amplitude interictal discharges were never associated with a significant Ca^2+^ change in astrocytes. This Ca^2+^ elevation and the following release of gliotransmitters may contribute to the maintenance of AP bursts and to the process of neuronal recruitment that characterize seizure discharge propagation. Our finding that the duration of the ictal discharges was significantly reduced upon inhibition of the astrocyte Ca^2+^ signal by bath perfusion with MPEP or PPADS is consistent with this hypothesis, which needs, however, to be specifically addressed in future experiments.

In the present study, we also addressed a possible role of the late astrocyte response in the propagation of the ictal discharge outside the focal region. After BAPTA introduction in field B astrocyte syncytium, the ictal discharge still propagated to this region and further, suggesting that Ca^2+^ elevations in field B astrocytes may have no role in this process. Given that initiation, propagation and cessation of the ictal discharge are likely governed by distinct mechanisms [3], it would not be surprising that astrocytes have, indeed, a role in ictal discharge initiation but not in propagation. This conclusion is, however, reasonable, but it is not proven beyond all doubt. Indeed, the inhibition by BAPTA could be exerted only in astrocytes from a small sector of the large field B, whereas astrocytes outside this sector were totally unaffected. Their activation might thus be sufficient to sustain the propagation of the ictal discharge even to the small sector where astrocytes were inhibited by BAPTA. As to MPEP/PPADS, when locally applied to field B, these competitive receptor antagonists failed to inhibit the ictal discharge propagating to this region. These results, however, do not allow us to draw any conclusions since the ictal discharge invading field B still activated a significant response in astrocytes even in the presence of MPEP/PPADS. To clarify this point, another experimental approach is thus required.

It is unclear why MPEP/PPADS failed to inhibit the Ca^2+^ elevation evoked by the ictal discharge in field B astrocytes. It is likely that, with respect to the NMDA pulse, the ictal discharge represents a more powerful stimulus that triggers the release of glutamate and ATP. Accordingly, the extracellular concentration of MPEP/PPADS reached after local applications might have been insufficient to inhibit the large activation of astrocyte mGlu and P2 receptors upon the ictal discharge. However, mechanisms other than mGlu and P2 receptor activation may be also involved in this astrocyte response.

Interictal discharges failed to activate significantly a Ca^2+^ elevation in astrocytes. Recently, it has been reported that glutamate release triggered by Ca^2+^ elevations in astrocytes plays a predominant, if not obligatory role in the generation of epileptic activity in the hippocampus and, in particular, in the slow depolarization shift associated with interictal discharges [9]. This conclusion is, however, at variance with a number of studies showing that both interictal and ictal seizure-like discharges from different brain regions, including the hippocampus, are strictly linked to neuronal activity being efficiently prevented or blocked, depending on the time of application, by TTX [10],[38],[56]–[58]. In the present study, we observed that i) the interictal activity was not blocked after Ca^2+^ elevations in astrocytes were drastically reduced; and ii) synchronous astrocyte Ca^2+^ elevations were never observed to accompany an interictal discharge in the different models. We thus failed to confirm a role of astrocytic glutamate in interictal discharge generation. The reasons for this discrepancy are, at present, unknown.

### Conclusions and Perspectives

The present study reveals a crucial role of neuron–astrocyte interactions in sculpting activity at the epileptogenic zone. When a group of neurons is abnormally active (due to acquired or genetic causes), ictal epileptiform events may occur through the activation of astrocytes. Astrocytes can thus play a key role in seizure initiation in a nonepileptic brain tissue and, in contrast to previous observations [9], do not appear to be involved in the generation of the interictal events. This peculiarity makes the astrocyte–neuron unit a primary target for novel drug development aimed at interfering selectively with ictogenesis, without affecting the interictal activity that, by preventing seizure precipitation, may have a beneficial role in focal epilepsies [59],[60].

The high reproducibility in the generation of comparable ictal discharges represents an important advantage of our new EC slice model of ictogenesis. This model allowed us to investigate the early events that, at a restricted brain site, predispose neurons to seizure and to obtain some insights into the mechanism of focal ictal generation that involves astrocytes. Other aspects that were not addressed in the present study, such as the neuronal recruitment process during the diffusion of the ictal discharge to regions distant from the site of ictal discharge generation, could be investigated in this model. These acute experiments set the conditions for validating the mechanisms here described in future studies in chronic models of epilepsy, including genetically determined in vivo models of epilepsy, that more closely mimic the complex feature of seizures in epileptic patients. A validation of the astrocyte role in seizures generation in these models is fundamental to provide further arguments in favour of astrocytes as targets for developing new therapeutic strategies for epilepsies.

## Materials and Methods

### Ethics Statement

All experimental procedures were authorized by the Italian Ministry of Health.

### Brain Slice, Guinea Pig Brain Preparations, and Dye Loading

Transverse cortico-hippocampal slices were prepared from postnatal day 14–18 Wistar rats or pGFAP-EGFP transgenic mice [61], and loaded with OGB1-AM (excited at 488 nm) or Rhod-2 (excited at 543 nm), respectively, as previously described [8]. Briefly, brain was removed and transferred to ice-cold cutting solution containing (in mM): NaCl, 120; KCl, 3.2; KH_2_PO_4_, 1; NaHCO_3_, 26; MgCl_2_, 2; CaCl_2_, 1; glucose, 10; Na-pyruvate, 2; and ascorbic acid, 0.6; at pH 7.4 (with 5% CO_2_/95% O_2_). Coronal slices were obtained by cutting with a Leica vibratome VT1000S in the presence of the ionotropic glutamate receptor inhibitor kynurenic acid (2 mM). Slices were recovered for 15 min at 37°C and then loaded with the Ca^2+^-sensitive dye OGB1-AM (Invitrogen) for 60 min at 37°C. Loading was performed in the cutting solution containing sulfinpyrazone (200 µM), pluronic (0.12%), and kynurenic acid (1 mM). After loading, slices were recovered and kept at room temperature in the presence of 200 µM sulfinpyrazone. Brains from postnatal day 14–20 guinea pigs were isolated and perfused at a rate of 5.5 ml/min through the basilar artery [19],[62] with a solution containing (in mM): NaCl, 126; KCl, 3; KH_2_PO_4_, 1.2; MgSO_4_, 1.3; CaCl_2_, 2.4; NaHCO_3_, 26; glucose, 15; and 3% dextran M.W. 70.000; oxygenated with a 95% O_2_/5% CO_2_ gas mixture (pH 7.3). The dye OGB1-AM (50 µg) was diluted in 5 µl of standard pluronic/DMSO solution and 75 µl of saline, and filtered through a 0.2-µm microfilter (Millipore). A patch pipette (3–4 MΩ was used to pressure inject (1–2 min at 4 PSI) the Ca^2+^ dye into the EC at a depth of about 200 µm via a picospritzer (NPI Electronics). Following this procedure, the Ca^2+^ signal from astrocytes, neurons, and neuropile was monitored. All experiments were performed at 33–35°C.

### Ca^2+^ Imaging

In slice experiments, we used a TCS-SP2-RS or a TCS-SP5-RS confocal microscope (Leica) equipped with a 20× objective (NA, 1.0) and a CCD camera for differential interference contrast images. For experiments on isolated guinea pig brains, we used a Fluoview 300 scanning head customized for two-photon microscopy equipped with a 5W Verdi-Mira laser (Coherent) tuned at 830 nm and external photomultipliers (Hamamatsu). Time frame acquisitions from 314 ms to 1.24 s (with one to six line averaging) were used. No background subtraction or other manipulations were applied to digitized Ca^2+^ signal images that are reported as raw data, with the exception of the difference images in Figure 4 that were obtained by subtracting the prestimulation image from the poststimulation image. In brain slice preparations, neurons and astrocytes were distinguished on the basis of the distinct kinetics of their Ca^2+^ response to a stimulation with high K^+^ extracellular solution (40 mM) obtained by isosmotic replacement of Na^+^ with K^+^ [16], applied at the end of the recording session in the presence of 1 µM TTX (Figure S7). Due to the lack of voltage-dependent Ca^2+^ channels in astrocytes, the Ca^2+^ elevation in these cells upon high K^+^ stimulation occurs with a delay of several seconds with respect to the response in neurons, and appears to be mediated by glutamate release from depolarizing neurons [17]. In the present study, the presence of TTX was necessary to block the epileptic discharges and the underlying Ca^2+^ changes in neurons and astrocytes that would have hampered the possibility to distinguish these cells from their different responses to high K^+^ stimulation. Astrocytes were identified also by their small size, low membrane potentials (−74±0.4 mV without the correction for the liquid junction potential at the pipette tip, which was 15 mV; *n* = 9), and passive responses to a series of depolarizing steps. In slices from pGFAP-EGFP mice, astrocytes were identified by their green GFP fluorescence. In the guinea pig brain, astrocytes were identified using the astrocyte-specific marker sulforhodamine 101 (Invitrogen) applied at 100 µM to the cortical surface [63]. The onset of the slow Ca^2+^ elevation in astrocytes was determined on the basis of a threshold criterion. The onset was identified by the change in Δ*F*/*F*_0_ that should be more than two standard deviations over the average baseline and remained above this value in the successive frames for at least 2 s (two to six frames, depending on the frame acquisition rate).

### Electrophysiology and NMDA Pulse Applications

Rat brain slices in a submerged chamber (Warner Instruments) were continuously perfused at a rate of 2 ml/min with (in mM): NaCl, 120; KCl, 3.2; KH_2_PO_4_, 1; NaHCO_3_, 26; MgCl_2_, 1; CaCl_2_, 2; glucose, 10; sulfinpyrazone, 0.2; at pH 7.4 (with 95% O_2_/5% CO_2_). Whole-cell patch-clamp recordings in rat brain slices were performed using standard procedures and one or two Axopatch-200B amplifiers (Molecular Devices), as previously reported [8]. Typical pipette resistance was 3–4 MΩ for neurons. Data were filtered at 1 kHz and sampled at 5 kHz with a Digidata 1320 interface and pClamp8 software (Molecular Devices). Whole-cell intracellular pipette solution was (in mM): K-gluconate, 145; MgCl_2_, 2; EGTA, 0.5; Na_2_ATP, 2; Na_2_GTP, 0.2; HEPES, 10; to pH 7.2 with KOH, and contained a low concentration (10 µM) of OGB1 (Invitrogen); osmolarity, 305–315 mOsm. Data analysis was performed with Clampfit 8 and Origin 6.0 (Microcal Software) software. SICs with an amplitude greater than 20 pA and a rise time slower than 10 ms are classified as SICs, as described previously. SIC rise time was calculated with the 20%–80% criterion and the decay time as the time constant of a single exponential fit. The delay of each SIC activated in neurons after astrocyte stimulation with TFLLR was calculated with respect to the peak of the immediately preceding astrocyte Ca^2+^ increase. Interictal and ictal seizure-like events resembling those recorded at the electrographic recordings from patient's brain [21], at a cellular level manifest as intense and hypersynchronous discharges that involve large neuronal population and fundamentally differ in their duration. Despite this common characteristic, they have radically different durations. Indeed, the duration of the epileptic event was an important criterion for classifying interictal and ictal events in slice and the isolated whole guinea pig brain preparations. In Ca^2+^ imaging experiments, interictal events lasted less that 3 s, whereas ictal discharges were sustained for tens of seconds with a final pattern of highly synchronous afterdischarges. The duration of ictal events varied between 15 and 110 s in brain slices and between 21 and 152 s in the guinea pig brain. Postictal depression was also consistently observed after an ictal event, whereas it was not present after an interictal spike [21]. A pressure ejection unit (PDES, NPI Electronics) was used to apply pressure pulses (4–10 psi, 200–600 ms duration) to NMDA-containing pipettes. Pulse pressure (or duration) was increased until a double NMDA pulse evoked an ictal discharge. The stimulus parameters for successive stimulations remained unchanged over the entire recording session, except in the BAPTA experiments in which they were changed to increase the stimulation of neurons by NMDA and thus to recover the ictal discharge after inhibition of Ca^2+^ signals in astrocytes. In the double NMDA pulse, the interval between the two pulses was 3 s. NMDA pulses applied with intervals of 5 s, but not 20 s also triggered an ictal discharge (unpublished data). For BAPTA dialysis into the astrocyte syncytium, we used a patch pipette (5–6 MΩ; 310–315 mOsm) containing (in mM): K-methylsulfate, 50; ATP, 2; GTP, 0.4; HEPES, 10; BAPTA, 50. To avoid a leakage of BAPTA from the pipette during seal formation, the BAPTA solution was backfilled after loading the tip with a standard intracellular solution. Texas Red dye (excited at 543 nm) was included at 0.2 mM in a patch pipette containing standard solution and monitored 50–60 min after the whole-cell configuration. For the BAPTA and Texas Red experiments, only GluT (coupled) astrocytes were included. GluT (coupled) and GluR (uncoupled) astrocytes were distinguished according to their different responses to hyperpolarizing and depolarizing current pulses of increasing amplitude and 750 ms duration. Field potentials were recorded from the guinea pig brain with saline-filled micropipettes used to deliver OGB1-AM, via a multichannel differential amplifier (NPI Electronics). A precise allignement of Ca^2+^ and electrophysiological signals was achieved by acquiring with a syncronisation signal produced by the confocal microscope. Tip potential was measured against a ground reference placed in the recording chamber by means of a voltage follower coupled to an amplifier.

### Drugs

MPEP (50 µM), PPADS (10 µM), TTX (1 µM), D-AP5 (50 µM), 4-AP (100 µM; Ascent Scientific), TFLLR (10 µM; Tocris), and picrotoxin (50 µM; Sigma-Aldrich) were bath applied. TFLLR (1 mM) and NMDA (1 mM; Sigma-Aldrich) were pressure applied. To induce local astrocyte inhibition, we applied pressure pulses of 2 s per 5 min at a frequency of 0.1 Hz to a pipette containing MPEP (500 µM) and PPADS (5 mM). Bicuculline methiodide (50 µM; Sigma-Aldrich) was applied by arterial perfusion to guinea pig brains.

### Monte Carlo Simulation Procedures

The Monte Carlo simulation was designed to test whether the observed series of stimuli and ictal episodes were compatible with a random distribution. Each simulation run generated randomly distributed stimuli and ictal events based on i) recording duration; ii) number of stimuli and ictal events; iii) minimum interval between stimuli; iv) minimum interval between two successive ictal events; and v) minimum interval between a stimulus and an ictal event (ictal events seem to be followed by at least 20 s of refractory period). These rules imply that the occurrence of ictals and pulses are not completely independent. The random generator produced 30,000 temporal series for each experimental run, using experiment-specific parameters. Figure S2A shows an experiment and three simulated runs. The distance between each stimulus and the first following ictal events were computed for each simulation. The datasets were used to compute the density probability p(*t*) of observing one ictal at time *t* after an astrocyte activation (Figure S2B). The cumulative probability CP(*t*) is obtained by the integration of the probability density and yields the probability of observing an ictal at a time ≤*t* under the hypothesis that stimuli and ictals are not causally related (Figure S2C). Each ictal event in the experiment was associated with the delay from the immediately preceding stimulus, and the probability of observing the ictal was calculated. If the cumulative probability was less than 0.05, the event was deemed as not satisfying the null hypothesis. Results are presented in Figure S2D.

### Immunocytochemistry

Coronal slices (100-µm thick) of rat brains were cut with a VT 1000S vibratome (Leica) and directly fixed for 1 h in iced 4% paraformaldehyde in phosphate-buffered solution (PBS). Floating sections were first preincubated in a blocking solution (BS; 1% BSA, 2% horse serum, and 2% goat serum) containing 0.3% triton X-100 and subsequently incubated with the primary mouse anti-thrombin receptor PAR-1 antibody (1∶300, Zymed Laboratories, Invitrogen) and rabbit anti-GFAP (1∶500, Dako) diluted in BS. After 24 h, slices were washed at 4°C in PBS and incubated with the secondary antibodies (Cy3 conjugated donkey anti-mouse IgG, and Cy2 conjugated goat anti-rabbit F(ab')2 fragments; Chemicon International) for 2 h at room temperature. Slices were extensively washed in PBS, mounted in Elvanol, and observed with a Leica SP2 laser scanning confocal microscopy. Negative controls were performed in the absence of the primary antibodies. Images were assembled using CS Adobe Photoshop software.

### Data Analysis

The Ca^2+^ signal is reported as Δ*F*/*F*_0_, where *F*_0_ is the baseline fluorescence. Data are shown as mean ± standard error of the mean (S.E.M.). Unless stated otherwise, the Student *t*-test was used, with *p* values ≤0.05 taken as statistically significant.

## Supporting Information

Figure S1 **Interictal events activate in astrocytes only an increase in Ca^2+^ oscillation frequency.** (A) Representative experiment from a rat hippocampal slice showing the Ca^2+^ elevations in CA3 neurons (black trace, averaged signal from all 20 neurons monitored) corresponding to interictal discharges in the picrotoxin/zero-Mg^2+^ model. This interictal discharge activity is accompanied by an increase in Ca^2+^ oscillation frequency in astrocytes (blue traces). (B) Bar graphs reporting the mean astrocyte Ca^2+^ oscillation frequency in controls and during interictal activity. *p<0.05. (C) 2P-LSM images from the EC of a guinea pig brain before (*t*_0_), during (*t*_1_), and after (*t*_2_) an interictal discharge induced by arterial perfusion with bicuculline. Astrocytes (white arrowheads), neuropile (dashed circle), and a neuron (yellow arrow) are indicated. Scale bar represents 20 µm. (D) Field potential recording of two interictal discharges and correlated Ca^2+^ changes in the neuropile and the neuron indicated in (C). No correlated Ca^2+^ changes were observed from the two astrocytes (arrowheads in [C]).
(0.72 MB TIF)

Figure S2 **Analysis by Monte Carlo simulation.** (A) Diagram representing the entire length of the recording of the experiment partially reported, in terms of Ca^2+^ signal changes in neurons and astrocytes, in Figure 3F. Horizontal bar on the top marks the timing of the traces shown in Figure 3F. Black bars indicate the peak of the astrocyte Ca^2+^ response triggered by TFLLR stimuli, and the green boxes correspond to the ictal events. The three underlying rows represent three results from the Monte Carlo simulation procedure (see Materials and Methods). (B and C) Density probability (B) and cumulative probability (C) computed from the Monte Carlo simulation for the depicted experiment in (A). Green arrowheads indicate the *p* value for the five ictal events occurring during the recording. These are the probabilities for each ictal to be independent from the astrocyte activation. The value 1 − *p* (reported in [D]) is the probability of correlation of the timing of the ictal discharge with the astrocyte Ca^2+^ increase. (D) Graph reporting the probability that the 15 ictal events (black dots) observed in six experiments are correlated positively with an astrocyte Ca^2+^ increase induced by TFLLR in the picrotoxin/zero-Mg^2+^ model.
(0.35 MB TIF)

Figure S3 **In the picrotoxin/zero-Mg^2+^ model, a single NMDA application is sufficient to trigger epileptiform discharges.** Representative experiment showing the effect of single local NMDA stimulation (arrowheads) on neurons from an EC slice perfused with picrotoxin/zero-Mg^2+^. The Ca^2+^ signal from a neuron in the region close to the NMDA pipette tip (blue trace) and the current-clamp recording from a neuron located in a region distant from the NMDA pipette (black trace) revealed that NMDA puffs could induce a local response that either remained restricted (open arrowheads) or triggered a response (black arrowheads) that evolved into an interictal (second and third puffs) or an ictal event (fifth puff). Spontaneous and evoked interictal and spontaneous and evoked ictal discharges recorded from the patched neuron are undistinguishable (see lower panels). This observation validates our model since it suggests that both events i) are sustained by a similar number and subtype of active cells; and ii) rely on a common basic mechanism.
(0.46 MB TIF)

Figure S4 **The ictal discharge triggered by a local neuronal stimulation is highly reproducible.** (A) Pair recordings showing that successive ictal discharges occur in both field A and B neurons, whereas only field A neurons showed a direct NMDA effect. (B) Mean duration of successive ictal discharges evoked by repetitive double NMDA pulses expressed as relative values with respect to the first two ictal episodes. (C) Number of responsive neurons and mean Ca^2+^ change after repetitive double NMDA pulses applied in the presence of TTX (*n* = 3). Data are expressed as percentage of the first response.
(0.53 MB TIF)

Figure S5 **Spatiotemporal profile of the Ca^2+^ signal in neurons and astrocytes during a focal ictal discharge.** (A) Pseudocolour raster plots showing the time derivative of the smoothed fluorescence signal (by averaging over five points) for individual neurons and astrocytes from the region within 200 µm of the NMDA pipette tip (field A) and from the surrounding region (field B) during an ictal discharge evoked by a double NMDA pulse applied at *t* = 0. If the activation were propagating as a concentric wave originating at the focus, the time of ictal onset in each cell would increase proportionally with the distance from the focus. Therefore, the raster plots would show a diagonal band of activation. Instead, the development and spreading of the ictal discharge is more similar to a process of modular recruitments of groups of neurons (and astrocytes): cells in the field A enter in the ictal phase more or less simultaneously. Cells in field B are recruited simultaneously, but at a later time than cells at the focal site of activation. (B) Bar graphs showing the distribution of the Ca^2+^ elevation onsets in neurons and astrocytes. The onset of the Ca^2+^ signal for each neuron was defined as the time of the absolute maximum derivative value (that better reflects the large Ca^2+^ rise of the recruitment of neurons into the ictal discharge), whereas for astrocytes, it was the time of the first local maximum derivative value (that reflects the initial Ca^2+^ rise in these cells).
(1.76 MB TIF)

Figure S6 **BAPTA-containing astrocyte syncytium in field B does not impair ictal discharge generation.** The introduction of BAPTA into astrocytes from a sector of field B does not impair either the generation of the ictal discharge in field A or the engagement of distant neurons from fields B and C into the ictal discharge. Note, however, that in the BAPTA-containing region, field B astrocytes are still activated by the ictal discharge, although their Ca^2+^ response have reduced amplitude and slow kinetics.
(0.27 MB TIF)

Figure S7 **High-potassium stimulation as a tool for cell classification in brain slices.** (A) Pseudocolour image from an EC slice loaded with OGB1-AM. Scale bar represents 20 µm. (B) Ca^2+^ signal from the eight cells indicated in (A) during an ictal event in the 4-AP model (left traces) and during the perfusion with a 40 mM K^+^ solution in 1 µM TTX (right traces). TTX was perfused for 5 min to block the epileptic activity before perfusion with the high K^+^ solution. Note that presumed neurons, i.e., large cells 1–4, in response to high K^+^ displayed a Ca^2+^ elevation largely before that of the presumed astrocytes, i.e., small cells 5–8. This delayed Ca^2+^ elevation is due to the lack of voltage-dependent Ca^2+^ channels in these cells. (C) Pseudocolour image from an EC slice loaded with OGB1-AM from a different experiment on the picrotoxin/zero-Mg^2+^ model. Scale bar represents 20 µm. (D) Ca^2+^ signal from the eight cells indicated in (C) during an ictal event that arose spontaneously (left traces) and during the perfusion with a 40 mM K^+^ solution and TTX (1 µM; right traces). Note that similar to what observed in (B), presumed astrocytes, i.e., small cells 5–8, displayed a delayed Ca^2+^ response to the high-potassium stimulation with respect to that in the large cells, presumed neurons. Note the high synchronous Ca^2+^ peaks in neurons from both experiments that reflect the afterdischarges of the seizure-like event.
(1.80 MB TIF)

Video S1 **Response of astrocytes to ictal and interictal discharges in EC slices.** This video shows the synchronous Ca^2+^ increase in EC neurons after slice perfusion with picrotoxin/zero-Mg^2+^ that reflects an interictal and an ictal discharge (as reported in Figure 1A and 1B). Note that at the beginning of the video there is an interictal discharge that activates only one astrocyte. A subsequent ictal discharge evokes a large and synchronous Ca^2+^ response in most astrocytes (indicated by the arrowheads in the first frame). In the final part of the video, synchronous, repetitive Ca^2+^ elevations in neurons reflect the afterdischarges typical of the late ictal phase. Time frame, 314 ms.
(3.34 MB MOV)

Video S2 **Selective stimulation of astrocytes with TFLLR triggers an ictal discharge.** This video shows the Ca^2+^ change in neurons and in astrocytes (indicated in the first frame by the orange arrowheads) evoked by a local application of TFLLR in an EC slice during perfusion with picrotoxin/zero-Mg^2+^ (as reported in Figure 3F). TFLLR induces a Ca^2+^ increase in astrocytes that is followed by an ictal discharge. Time frame, 1 s.
(5.49 MB MOV)

Video S3 **A single NMDA stimulation triggers only a local Ca^2+^ response.** This video shows the Ca^2+^ increase that is restricted to a group of layer V-VI EC neurons that follows a single NMDA stimulation (as reported in Figure 4). The orange circle marks the timing of the NMDA puff and the position of the NMDA pipette. Note the absence of astrocyte responses. Time frame, 356 ms.
(0.78 MB MOV)

Video S4 **Two successive NMDA applications trigger an ictal discharge.** This video shows an ictal event generated by an NMDA stimulation composed of two successive puffs (as reported in Figure 4). The orange circle in field A marks the timing of the NMDA puffs and the position of the NMDA pipette, whereas the yellow circle in field B marks the engagement of neurons from this region into the ictal discharge. The orange arrowheads indicate astrocytes in field A at the onset of their Ca^2+^ increase. Note that Ca^2+^ elevations in these astrocytes accompany the development of the ictal discharge. Yellow arrowheads indicate astrocytes in field B (at the onset of their Ca^2+^ increase) that are activated after the emergence of the ictal discharge in field B. Time frame, 356 ms.
(2.39 MB MOV)

## Abbreviations

AP: action potential
EC: entorhinal cortex
2P-LSM: two-photon laser scanning microscopy
SE: status epilepticus
SIC: slow inward current
