# Supplementary figures and images for: An Excitatory Loop with Astrocytes Contributes to Drive Neurons to Seizure Threshold
Source: PLoS Biol. 2010 Apr 13;8(4):e1000352. doi: 10.1371/journal.pbio.1000352 (PMC2854117; doi:10.1371/journal.pbio.1000352)

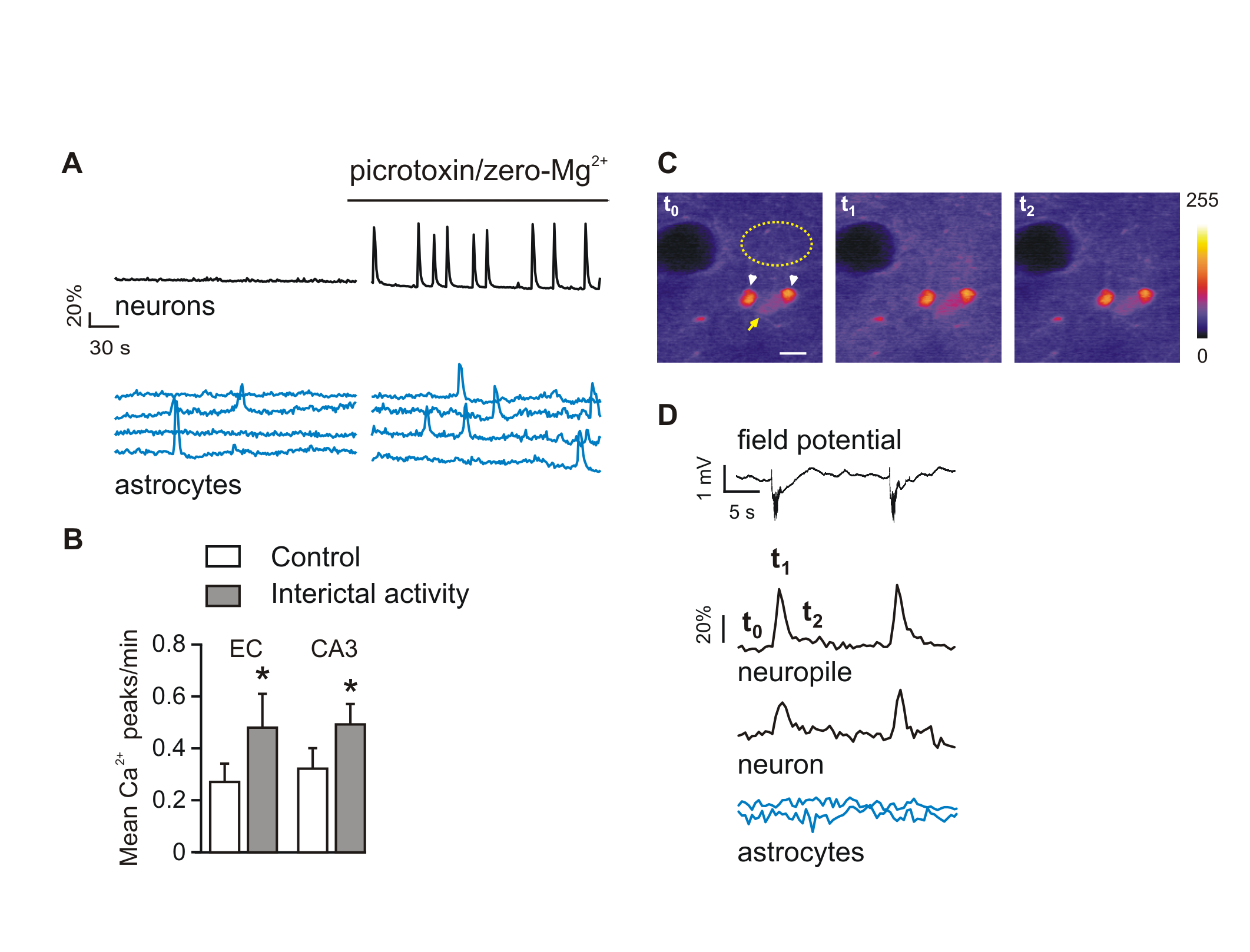

Supplement: Figure S1 — Interictal events activate in astrocytes only an increase in Ca2+ oscillation frequency. (A) Representative experiment from a rat hippocampal slice showing the Ca2+ elevations in CA3 neurons (black trace, averaged signal from all 20 neurons monitored) corresponding to interictal discharges in the picrotoxin/zero-Mg2+ model. This interictal discharge activity is accompanied by an increase in Ca2+ oscillation frequency in astrocytes (blue traces). (B) Bar graphs reporting the mean astrocyte Ca2+ oscillation frequency in controls and during interictal activity. *p<0.05. (C) 2P-LSM images from the EC of a guinea pig brain before (t 0), during (t 1), and after (t 2) an interictal discharge induced by arterial perfusion with bicuculline. Astrocytes (white arrowheads), neuropile (dashed circle), and a neuron (yellow arrow) are indicated. Scale bar represents 20 µm. (D) Field potential recording of two interictal discharges and correlated Ca2+ changes in the neuropile and the neuron indicated in (C). No correlated Ca2+ changes were observed from the two astrocytes (arrowheads in [C]). (0.72 MB TIF) [file pbio.1000352.s001.tif]

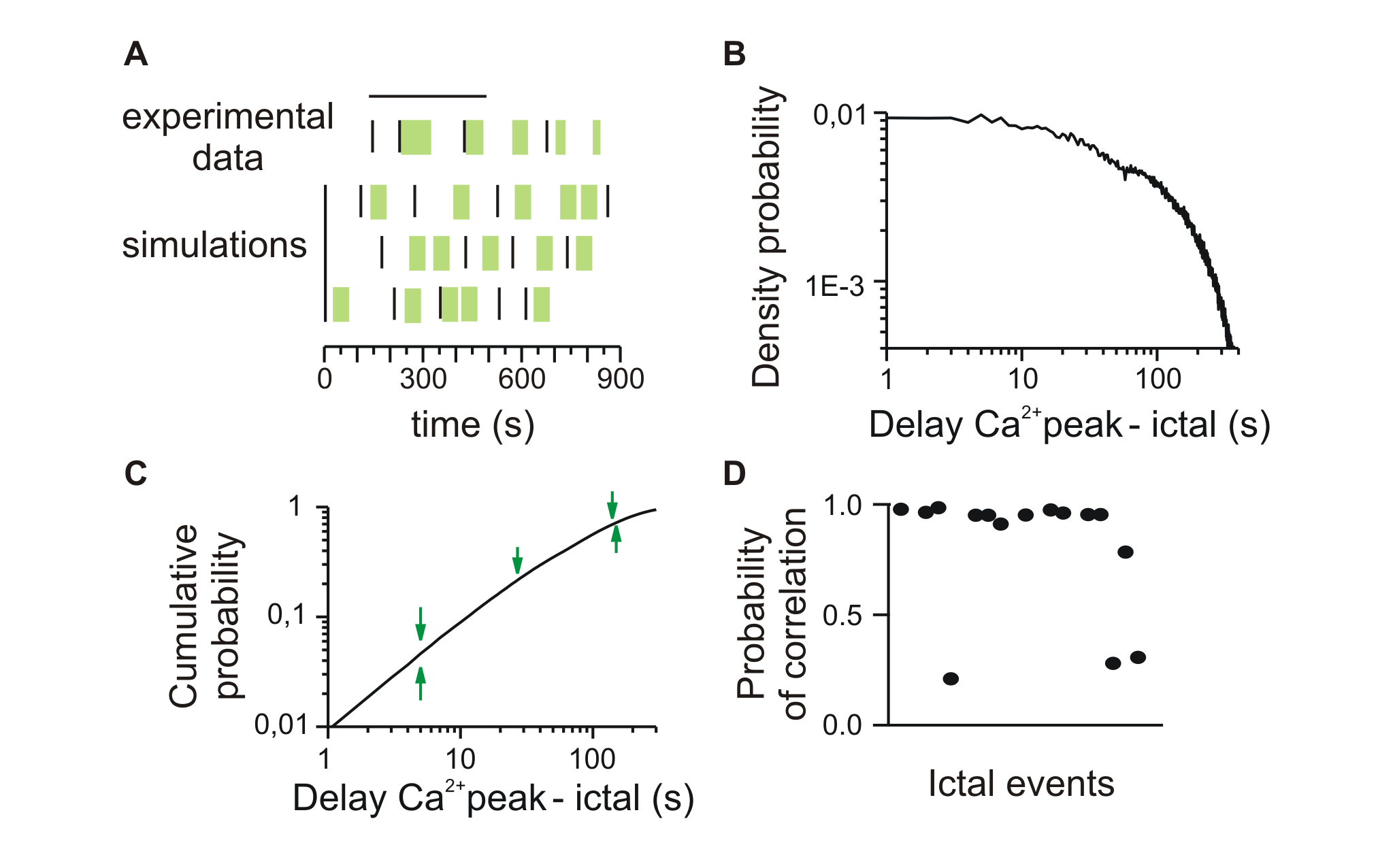

Supplement: Figure S2 — Analysis by Monte Carlo simulation. (A) Diagram representing the entire length of the recording of the experiment partially reported, in terms of Ca2+ signal changes in neurons and astrocytes, in Figure 3F. Horizontal bar on the top marks the timing of the traces shown in Figure 3F. Black bars indicate the peak of the astrocyte Ca2+ response triggered by TFLLR stimuli, and the green boxes correspond to the ictal events. The three underlying rows represent three results from the Monte Carlo simulation procedure (see Materials and Methods). (B and C) Density probability (B) and cumulative probability (C) computed from the Monte Carlo simulation for the depicted experiment in (A). Green arrowheads indicate the p value for the five ictal events occurring during the recording. These are the probabilities for each ictal to be independent from the astrocyte activation. The value 1 − p (reported in [D]) is the probability of correlation of the timing of the ictal discharge with the astrocyte Ca2+ increase. (D) Graph reporting the probability that the 15 ictal events (black dots) observed in six experiments are correlated positively with an astrocyte Ca2+ increase induced by TFLLR in the picrotoxin/zero-Mg2+ model. (0.35 MB TIF) [file pbio.1000352.s002.tif]

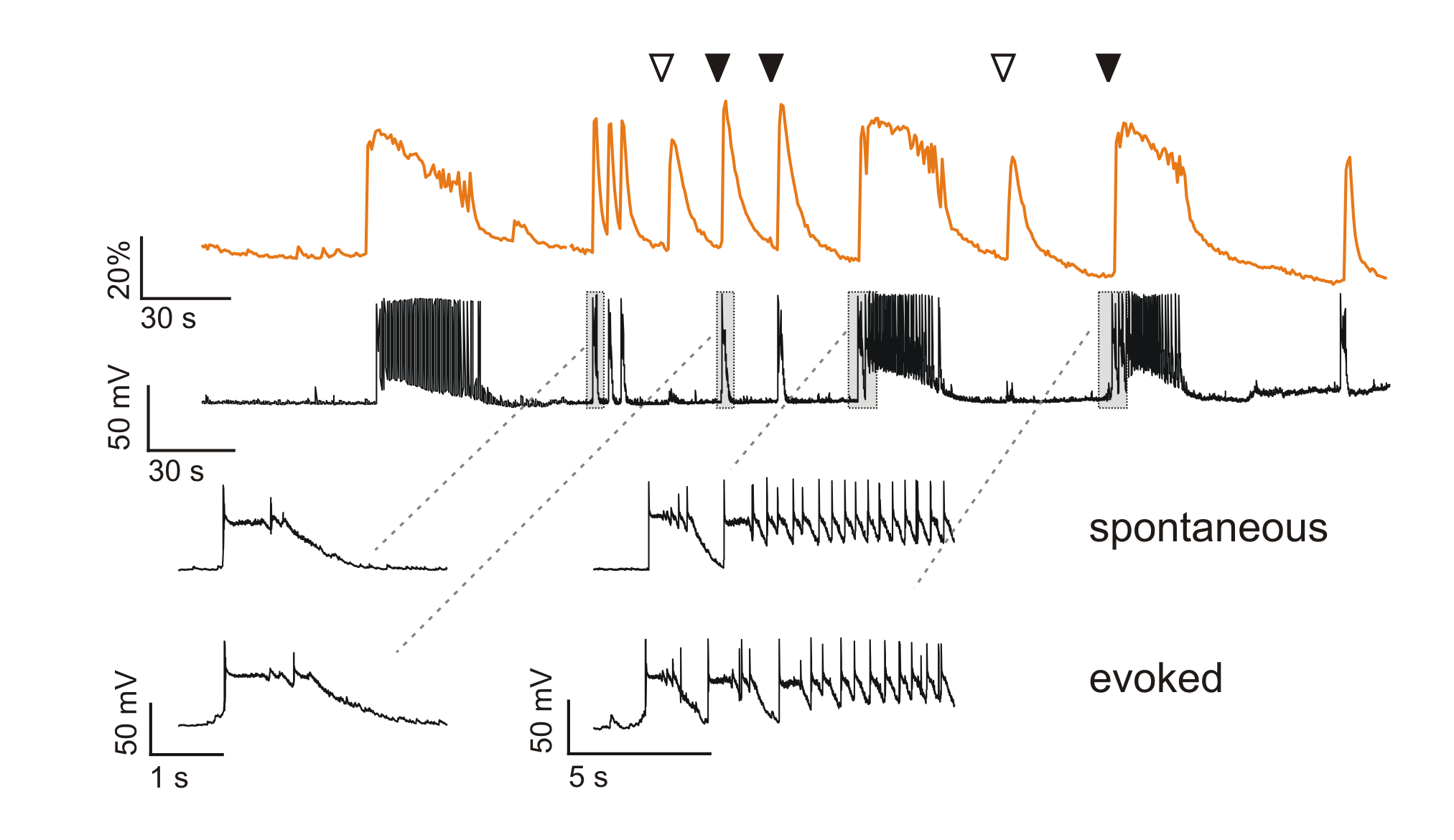

Supplement: Figure S3 — In the picrotoxin/zero-Mg2+ model, a single NMDA application is sufficient to trigger epileptiform discharges. Representative experiment showing the effect of single local NMDA stimulation (arrowheads) on neurons from an EC slice perfused with picrotoxin/zero-Mg2+. The Ca2+ signal from a neuron in the region close to the NMDA pipette tip (blue trace) and the current-clamp recording from a neuron located in a region distant from the NMDA pipette (black trace) revealed that NMDA puffs could induce a local response that either remained restricted (open arrowheads) or triggered a response (black arrowheads) that evolved into an interictal (second and third puffs) or an ictal event (fifth puff). Spontaneous and evoked interictal and spontaneous and evoked ictal discharges recorded from the patched neuron are undistinguishable (see lower panels). This observation validates our model since it suggests that both events i) are sustained by a similar number and subtype of active cells; and ii) rely on a common basic mechanism. (0.46 MB TIF) [file pbio.1000352.s003.tif]

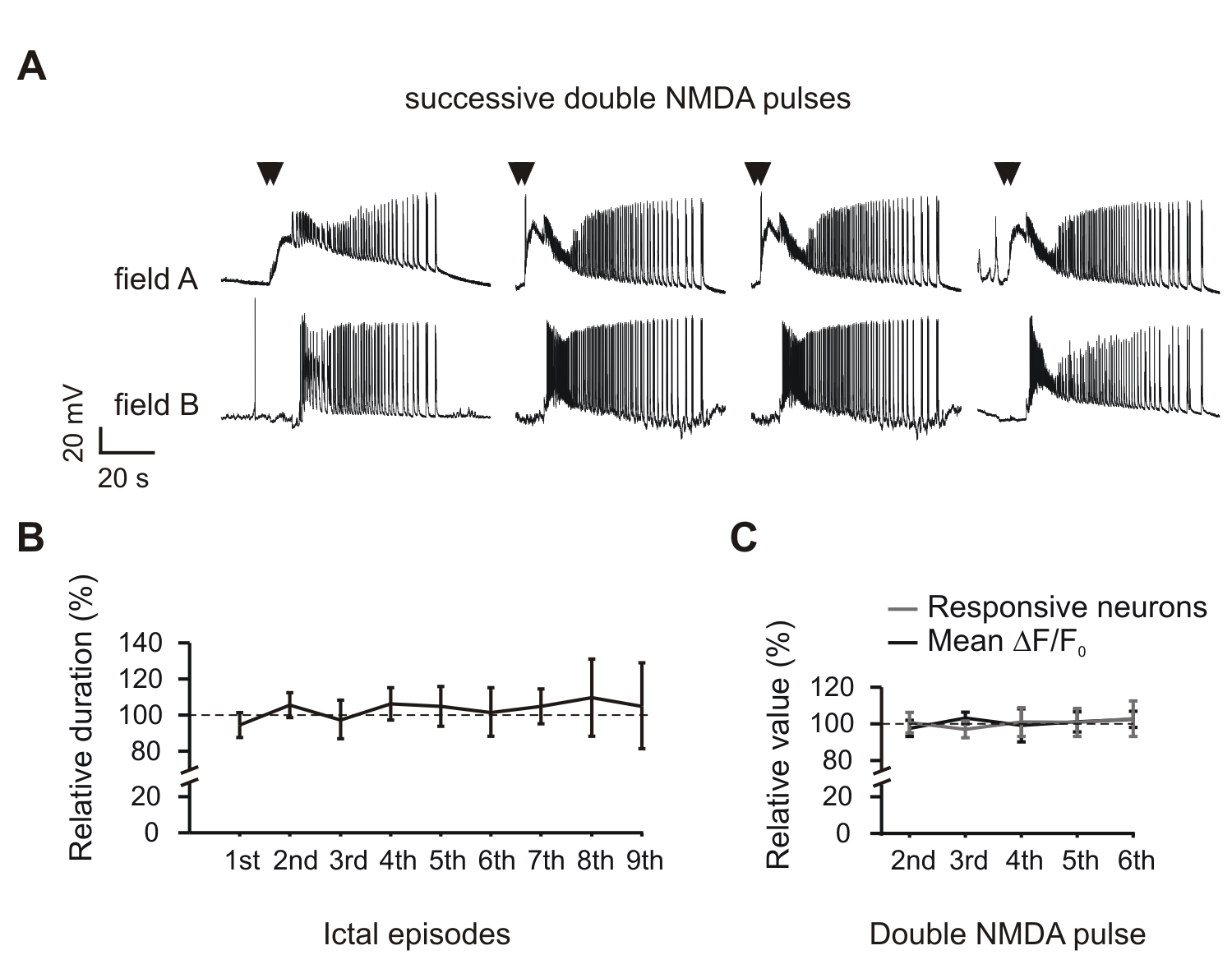

Supplement: Figure S4 — The ictal discharge triggered by a local neuronal stimulation is highly reproducible. (A) Pair recordings showing that successive ictal discharges occur in both field A and B neurons, whereas only field A neurons showed a direct NMDA effect. (B) Mean duration of successive ictal discharges evoked by repetitive double NMDA pulses expressed as relative values with respect to the first two ictal episodes. (C) Number of responsive neurons and mean Ca2+ change after repetitive double NMDA pulses applied in the presence of TTX (n = 3). Data are expressed as percentage of the first response. (0.53 MB TIF) [file pbio.1000352.s004.tif]

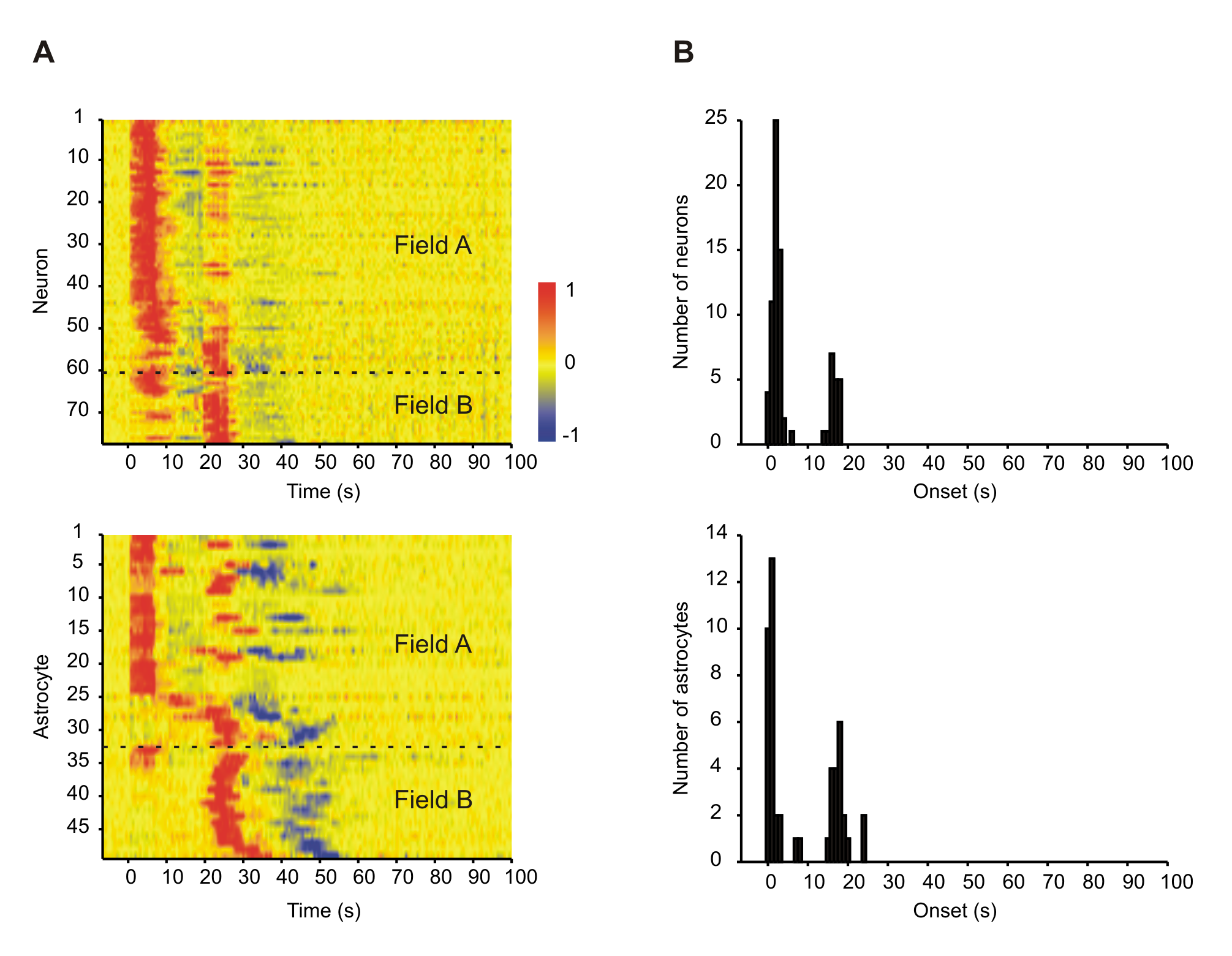

Supplement: Figure S5 — Spatiotemporal profile of the Ca2+ signal in neurons and astrocytes during a focal ictal discharge. (A) Pseudocolour raster plots showing the time derivative of the smoothed fluorescence signal (by averaging over five points) for individual neurons and astrocytes from the region within 200 µm of the NMDA pipette tip (field A) and from the surrounding region (field B) during an ictal discharge evoked by a double NMDA pulse applied at t = 0. If the activation were propagating as a concentric wave originating at the focus, the time of ictal onset in each cell would increase proportionally with the distance from the focus. Therefore, the raster plots would show a diagonal band of activation. Instead, the development and spreading of the ictal discharge is more similar to a process of modular recruitments of groups of neurons (and astrocytes): cells in the field A enter in the ictal phase more or less simultaneously. Cells in field B are recruited simultaneously, but at a later time than cells at the focal site of activation. (B) Bar graphs showing the distribution of the Ca2+ elevation onsets in neurons and astrocytes. The onset of the Ca2+ signal for each neuron was defined as the time of the absolute maximum derivative value (that better reflects the large Ca2+ rise of the recruitment of neurons into the ictal discharge), whereas for astrocytes, it was the time of the first local maximum derivative value (that reflects the initial Ca2+ rise in these cells). (1.76 MB TIF) [file pbio.1000352.s005.tif]

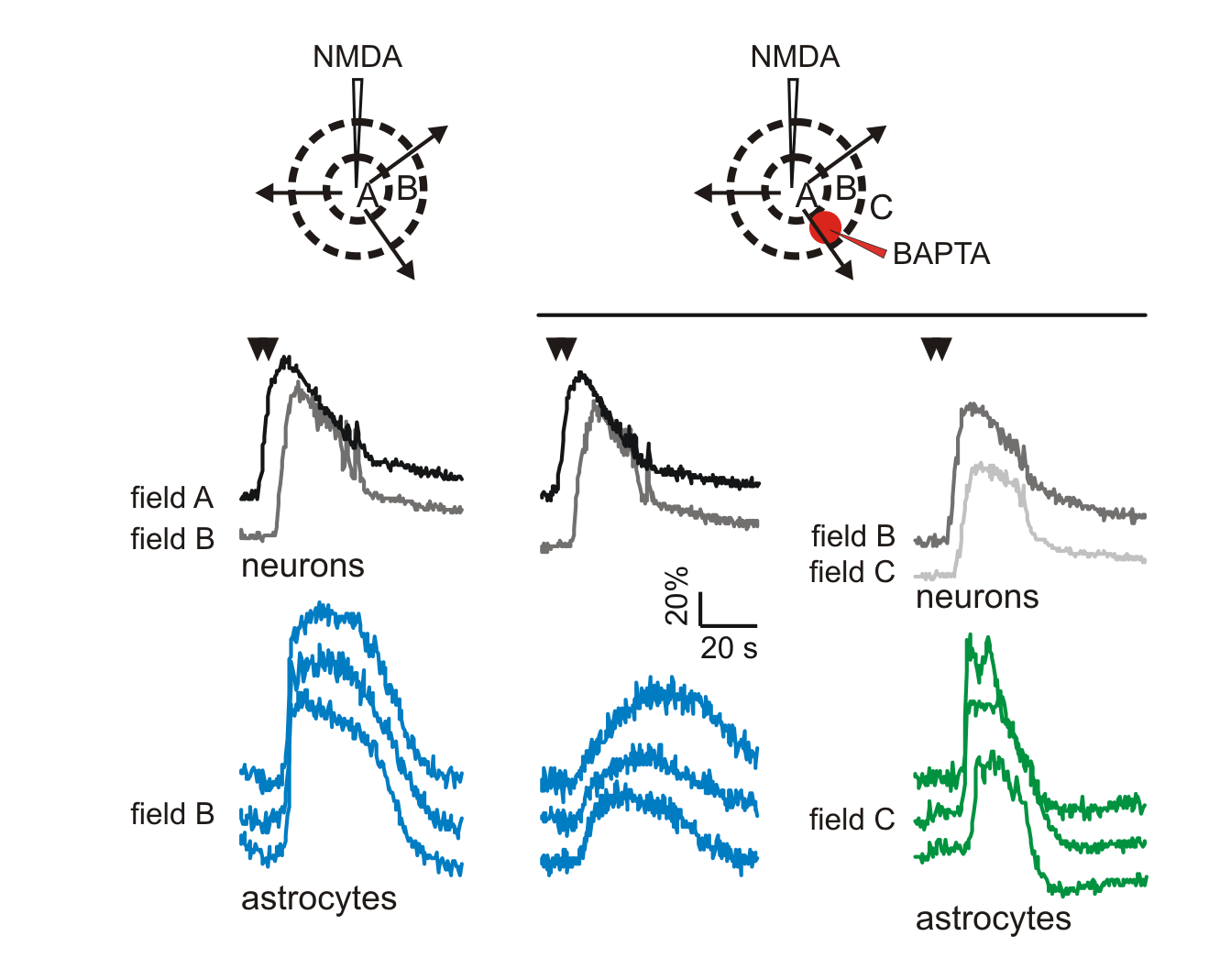

Supplement: Figure S6 — BAPTA-containing astrocyte syncytium in field B does not impair ictal discharge generation. The introduction of BAPTA into astrocytes from a sector of field B does not impair either the generation of the ictal discharge in field A or the engagement of distant neurons from fields B and C into the ictal discharge. Note, however, that in the BAPTA-containing region, field B astrocytes are still activated by the ictal discharge, although their Ca2+ response have reduced amplitude and slow kinetics. (0.27 MB TIF) [file pbio.1000352.s006.tif]

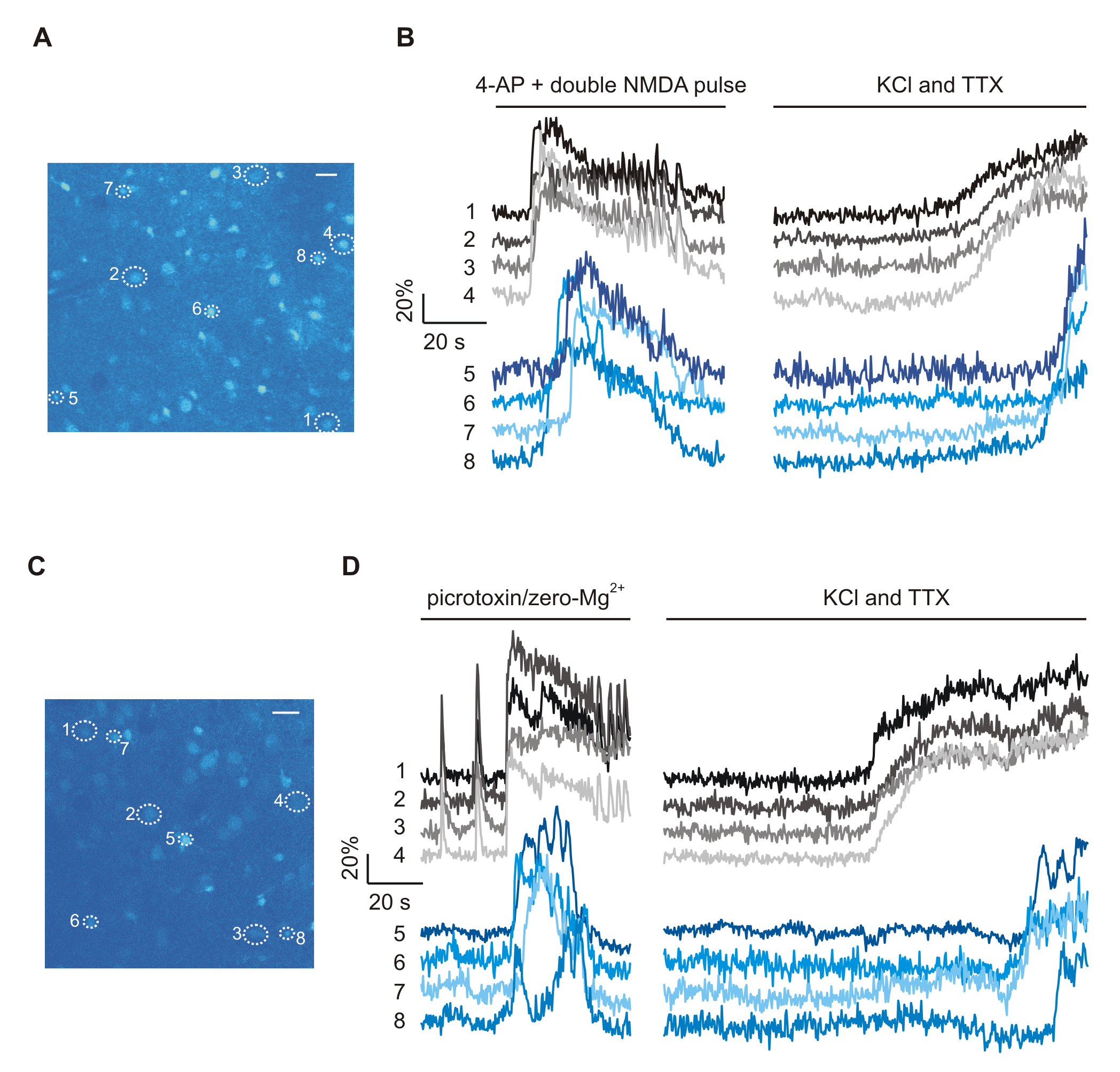

Supplement: Figure S7 — High-potassium stimulation as a tool for cell classification in brain slices. (A) Pseudocolour image from an EC slice loaded with OGB1-AM. Scale bar represents 20 µm. (B) Ca2+ signal from the eight cells indicated in (A) during an ictal event in the 4-AP model (left traces) and during the perfusion with a 40 mM K+ solution in 1 µM TTX (right traces). TTX was perfused for 5 min to block the epileptic activity before perfusion with the high K+ solution. Note that presumed neurons, i.e., large cells 1–4, in response to high K+ displayed a Ca2+ elevation largely before that of the presumed astrocytes, i.e., small cells 5–8. This delayed Ca2+ elevation is due to the lack of voltage-dependent Ca2+ channels in these cells. (C) Pseudocolour image from an EC slice loaded with OGB1-AM from a different experiment on the picrotoxin/zero-Mg2+ model. Scale bar represents 20 µm. (D) Ca2+ signal from the eight cells indicated in (C) during an ictal event that arose spontaneously (left traces) and during the perfusion with a 40 mM K+ solution and TTX (1 µM; right traces). Note that similar to what observed in (B), presumed astrocytes, i.e., small cells 5–8, displayed a delayed Ca2+ response to the high-potassium stimulation with respect to that in the large cells, presumed neurons. Note the high synchronous Ca2+ peaks in neurons from both experiments that reflect the afterdischarges of the seizure-like event. (1.80 MB TIF) [file pbio.1000352.s007.tif]
